# Perceptions of anomie in society shape support for wealth redistribution

**DOI:** 10.1111/bjso.70067

**Published:** 2026-03-29

**Authors:** Kelly Kirkland, Christoph Klebl, Christian T. Elbæk, Jolanda Jetten

**Affiliations:** ^1^ University of Queensland Brisbane Australia; ^2^ University of Melbourne Melbourne Australia; ^3^ Aarhus University Aarhus Denmark

**Keywords:** anomie, disintegration, dysregulation, economic inequality, redistribution

## Abstract

Understanding the factors that influence support for wealth redistribution is essential to address growing economic divides around the world. We propose that perceptions of anomie—the belief that society's social and political fabric is crumbling—can influence support for redistribution in opposing ways. When people see society as deteriorating, they may seek drastic change, increasing support for redistribution. Conversely, viewing society as descending into anomie may also foster a belief that the government will mismanage redistributed wealth, thereby reducing support. Study 1 examined these relationships in a U.S. sample, confirming the presence of these two opposing pathways, and Study 2 then replicated the findings in the UK. Study 3 tested this model experimentally, introducing the ‘anomie paradigm’ to explore how perceptions of anomie cause shifts in psychology. Here, participants were exposed to a fictitious society characterized by high or low anomie. The high (relative to low) anomie condition increased support for redistribution through a desire for change but simultaneously decreased support via concerns over government misuse. These findings highlight how perceptions of societal breakdown can shape redistributive preferences through co‐occurring psychological processes with opposing implications for policy support.

Economic inequality has intensified around the world in recent decades (Chancel et al., 2026; Piketty, 2020), a trend that has only deepened in the wake of the Covid‐19 pandemic (Chancel et al., 2022; Schifferes, 2020). Since 2020, the fortunes of the world's five wealthiest individuals have doubled, while nearly five billion people are relatively poorer today than they were before (Oxfam, 2024). Economic inequality has now reached such extreme levels that it rivals those observed at the height of Western imperialism in the early 20th century (Chancel et al., 2022). The extreme concentration of wealth within a small minority exerts substantial negative effects on both societal well‐being and individual behaviour. Extreme economic inequality has been associated with poorer health outcomes (Pickett & Wilkinson, 2015), increased crime (De Courson & Nettle, 2021; Fajnzylber et al., 2002), reduced social mobility (Chetty et al., 2014, 2016) and the loss of trust and cohesion between citizens (Elgar, 2010; Rothstein & Uslaner, 2005; Uslaner & Brown, 2005). It also contributes to a range of major societal crises, including the desire for strong and authoritarian leaders (Sprong et al., 2019), greater political polarization (Gu & Wang, 2022; Ordabayeva, 2019; Stewart et al., 2020) and environmental degradation (Chen et al., 2020). Addressing economic inequality is not only crucial for achieving fair outcomes and opportunities, but also essential for ensuring societal stability and well‐being (Jetten et al., 2021).

One of the most effective ways to reduce economic inequality is through redistributive policies, such as taxes on wealth and capital (Piketty, 2020; Zucman, 2014), which target the capital stock rather than just income (Saez & Zucman, 2019). Yet, public support for these policies can be low (Horowitz et al., 2020; Hoy & Mager, 2021). For example, only 60% of US Democrats and 20% of Republicans would say reducing economic inequality is a top priority for the government. Interestingly, preferences for such policies are quite elastic (e.g., Kuziemko et al., 2015) and robustly influenced by various factors, including political orientation (Armingeon & Weisstanner, 2022), one's own economic position (Brown‐Iannuzzi et al., 2021), cognitive biases (Walker et al., 2021) and perceptions of the magnitude of inequality (Knell & Stix, 2020; Norton & Ariely, 2011).

While a substantial body of literature has identified individual‐level factors that may influence support for redistribution (Ciani et al., 2021), it is equally important to consider broader societal conditions. We propose that the shared perception that society is descending into a state of *anomie* — defined as the breakdown of social integration and social regulation (Durkheim, 1897/1987; Teymoori et al., 2016, 2017) — may have nuanced consequences for support for redistributive policies. Specifically, perceptions of societal breakdown may give rise to multiple, potentially co‐occurring responses. On the one hand, a sense that society is crumbling may push people to seek drastic change (Kirkland et al., 2024; Klebl & Jetten, 2024; Salvador Casara et al., 2022; Sprong et al., 2019; Teymoori et al., 2017), thereby *increasing* support for redistribution. On the other hand, heightened perceptions of anomie may raise concerns about how effectively redistributive policies would be implemented, which may in turn *reduce* support for redistribution. This study aims to disentangle these potentially competing forces, providing insights into how perceptions of anomie relate to attitudes towards wealth redistribution.

## When societies break down

When individuals collectively feel that the norms and rules that once held communities together are unravelling, the very fabric of society begins to fray (Jetten et al., 2021; Kirkland et al., 2022; Salvador Casara et al., 2022; Sprong et al., 2019). This perception reflects the notion that society is descending into a state of anomie (Durkheim, 1897/1987; Teymoori et al., 2016, 2017). Anomie is not just an individual experience; it is a shared perception that emerges within a collective when a critical mass of people believes that societal norms and leadership are failing. Furthermore, it is not based on objective circumstances but on the *perception* of social decay, shaped through social communication and interaction (Teymoori et al., 2017). For example, even in communities where crime rates remain stable, a focus on isolated incidents in the media can create a shared sense that public safety is deteriorating.

Anomie consists of two key components: a perceived breakdown in both the social fabric (characterized by low trust and a lack of shared moral standards) and leadership (governing bodies of society are seen as illegitimate and ineffective), with these dimensions mutually reinforcing one another (Teymoori et al., 2016, 2017). This leads to a downward spiral, where the collapse of one pillar puts pressure on the other, resulting in a widespread perception of societal decay. This has important consequences for human psychology. As anomie takes hold, it leaves fundamental human needs unmet, such as the need for meaning, belonging, self‐esteem and control (Greenaway et al., 2016; Williams, 2009). In response, perceptions of anomie may activate multiple psychological processes aimed at coping with this breakdown. On the one hand, anomie can elicit a motivation to regain control through drastic societal change (Fritsche et al., 2011; Kirkland et al., 2024). At the same time, anomie may give rise to a sense of helplessness and resignation, in which individuals disengage from collective solutions because they perceive societal problems as beyond repair (Norasakkunkit & Uchida, 2011; Teymoori et al., 2017; Thorlindsson, 2004). The relative strength of these co‐occurring processes may vary across contexts, and together they help explain how perceptions of anomie shape support for efforts to redress economic inequality.

## Redressing inequality under anomie

The profoundly different reactions to anomie may have opposing implications for support for wealth redistribution. First, perceiving a state of anomie may *increase* support for wealth redistribution because anomie creates a sense of lost regulation and control at the societal level, triggering a motivation to reassert order and stability (Fritsche et al., 2011; Kirkland et al., 2024). Previous research demonstrates that heightened perceptions of anomie are associated with attitudes and behaviours aimed at restoring structure, clarity, and normative regulation within society (Kirkland et al., 2024; Salvador Casara et al., 2022; Sprong et al., 2019). Recent work further shows that perceptions of anomie undermine feelings of political control and increase uncertainty, thereby motivating support for system‐level arrangements that symbolically restore order and regulation (Neerdaels et al., 2024). Consistent with this perspective, individuals experiencing anomie may come to view drastic societal change as necessary to re‐establish clear rules and limits on an otherwise unregulated system (Klebl & Jetten, 2024). Extreme wealth inequality can serve as a salient signal that societal rules and constraints have broken down, particularly under conditions of perceived anomie. In this context, wealth redistribution may be especially appealing not merely as a policy preference, but as a symbolic means of reasserting collective regulation and moral order. Constraining extreme inequalities may help restore a sense that the social system is governed by shared rules and fairness norms.

At the same time, a perception of anomie may *diminish* support for wealth redistribution by fostering a sense of helplessness and disengagement towards collective solutions (Neerdaels et al., 2024; Teymoori et al., 2016, 2017). When societal problems are perceived as beyond repair, individuals may become skeptical that institutional action can meaningfully address inequality. In this context, the government may be seen as incapable of managing resources fairly or implementing reform effectively. Rather than viewing redistribution as a viable solution to inequality, perceptions of anomie may therefore foster concerns that it will only deepen corruption and benefit elites rather than the broader public. Thus, perceptions of anomie may simultaneously motivate increased support for redistribution as a pathway to restore order, while also activating skepticism that dampens support due to concerns about governmental misuse of resources. Understanding this dynamic may aid in developing more effective interventions to redress growing economic inequality in society.

## The current study

This work aims to examine the relationship between perceptions of anomie and support for wealth redistribution. Specifically, we sought to determine whether anomie might increase support for redistribution through a desire for drastic change, as well as decrease support through a belief that the government would mismanage redistributed wealth. To investigate these questions, we conducted two correlational studies using samples from the US (Study 1) and the UK (Study 2). Study 3 then introduced the ‘anomie paradigm’ where participants were exposed to a fictitious society characterized by high or low anomie, allowing us to test how societal decay causes changes in support for redistribution. Together, this work clarifies how perceptions of anomie shape attitudes towards redistribution and may inform efforts to address rising inequality.

## OPEN SCIENCE STATEMENT

All studies were preregistered on the Open Science Framework prior to data collection (Study 1: https://osf.io/4t5ke/overview; Study 2: https://osf.io/745zr/overview; Study 3: https://osf.io/pg7jr/overview).

Study 1 was preregistered as an experimental investigation of the effects of manipulating real‐world perceptions of anomie (high, low, control) on support for wealth redistribution. However, although the high anomie condition produced a small increase in perceived anomie relative to control, the low anomie condition did not reliably reduce perceived anomie, resulting in a weak and asymmetric manipulation (see Supplementary Materials S1, Figure S1). Because the preregistered experimental manipulation was not successful, the hypotheses were not tested. Instead, Study 1 was treated as exploratory and analysed using correlational methods.

Materials, data and analysis code for all studies can be accessed via the following link: https://osf.io/ayjzn/overview.

## STUDY 1

This study explored the link between perceptions of anomie and support for redistribution, as well as potential mechanisms. We proposed no hypotheses, and analyses were exploratory.

### Method

See Supplementary Materials S1 for the full list of items. While this study was exploratory, all measures were preregistered.

#### Participants

In total, 601 participants residing in the US took part in the study. The data was collected online from Prolific Academic using the platform's representative sampling tools, which apply demographic quotas based on U.S. Census benchmarks to approximate population distributions in age, sex, ethnicity and political affiliation. Rigorous attention checks were applied in the study, and 28 participants were removed for failing at least one of the five preregistered checks. The final sample was therefore 573 participants.

The sample was roughly equally split by sex (female = 295; male = 274) and participants were aged between 18 and 81 (*M* = 45.11, SD = 16.02). Participants were asked for their political orientation with two items on a scale from 1 (*left/progressive*) to 7 (*right/conservative*) and were centrist on average (*M* = 3.76, SD = 1.82). Participants identified as either Democrat (*n* = 173), Independent (*n* = 228) or Conservative (*n* = 168). The sample was also predominantly White (56.5%), followed by Black (12.6%), mixed race (11.5%), Asian (9.9%) or other ethnicity (8.7%). Finally, participants were given the MacArthur Scale of Subjective Social Status (Glei et al., 2018; Goodman et al., 2001; Singh‐Manoux et al., 2003). They were shown a 10‐rung ladder and asked to indicate where they felt they fit on the ladder relative to others, from 1 (*bottom rung/worst off in society*) to 10 (*top rung/best off in society*). On average, participants tended to perceive themselves as middle class (*M* = 5.08; SD = 1.67) and reported moderate importance of religion (*M* = 3.48; SD = 2.41) on a scale from 1 (*not at all important*) to 7 (*extremely important*).

#### Measures

Study 1 was designed to be exploratory, with the aim of examining the relationship between perceptions of anomie and support for wealth redistribution. To this end, we included a broad set of theoretically plausible variables that may be associated with this relationship. These measures were collected to allow for an open examination of potential psychological correlates and are reported in full for transparency.

##### Perceptions of anomie

Perceptions of anomie were assessed using 12 items (Teymoori et al., 2016), with six items measuring breakdown in the social fabric of society, for example, ‘People think that there are no clear moral standards to follow’ and six items measuring breakdown in leadership, for example, ‘Some laws are not fair.’ Responses were assessed on a scale ranging from 1 (*strongly disagree*) to 7 (*strongly agree*), with higher scores indicating greater perceptions of anomie. A total anomie score was obtained (*α* = .87), as well as a score for a breakdown in the social fabric of society (*α* = .80) and breakdown in leadership (*α* = .87).

##### Support for wealth redistribution

First, a general measure of support for wealth redistribution was measured with the item: ‘The United States government should take measures to reduce differences in wealth levels’, on a scale from 1 (*strongly disagree*) to 7 (*strongly agree*). This measure was sourced from the European Social Survey European Research Infrastructure (ESS ERIC) (2025), adapting this item to wealth rather than income. Second, a specific measure for support for redistribution was measured with the item: ‘How much would you be in favour of a wealth tax on the top 0.1%?’, on a scale from 1 (*strongly oppose*) to 7 (*strongly favour*; Elbæk et al., 2026).

##### Desire for drastic change

In line with past work (Klebl & Jetten, 2024), the need for drastic change was measured with four items (e.g., ‘We cannot go on like this and therefore have to change the system’), measured on a scale from 1 (*not at all*) to 7 (*very much so*). However, the one reverse coded item (e.g., ‘Quick fixes are possible and sufficient’) proved problematic (original *α* = .63) and was removed (Cortina, 1993).^1^ The remaining three items were averaged (*α* = .82).

##### Fears of government misuse

Participants were first told: ‘Imagine the American government started taxing the rich with the intention of giving that money to the poor. How much do you agree with the following statements:’ Two items were developed for this study to capture fears that the government would misuse redistributed funds. These were ‘We can't trust the government to use those funds correctly’ and ‘The government would misuse that money’, measured on a scale from 1 (*strongly disagree*) to 7 (*strongly agree*). Items were highly correlated (*r* = .85) and averaged.

##### Fears of misuse by the poor

Participants completed two items assessing concerns that people living in poverty would misuse redistributed funds: ‘We can't trust poor people to use those funds correctly’ and ‘Poor people would misuse that money.’ Responses were measured on a scale from 1 (*strongly disagree*) to 7 (*strongly agree*). Items were highly correlated (*r* = .85) and averaged.

##### Perceived fairness and evaluation of inequality

Perceived fairness of wealth disparities was assessed using two measures. First, participants completed four items capturing normative unfairness concerns regarding economic inequality, drawn from the Societal Inequality Scale (SIS; Schmalor & Heine, 2021). These items assessed the extent to which participants viewed high levels of inequality and unequal life chances as unjust (e.g., ‘It is extremely unfair if the overall amount of economic inequality is very high’). Responses were recorded on a scale from 1 (*strongly disagree*) to 7 (*strongly agree*), and items were averaged to form a composite score (*α* = .88). Second, participants completed a single‐item evaluation of wealth inequality, adapted from the International Social Survey Programme (ISSP): ‘Differences in wealth in the United States are too large’. Responses were recorded on the same 7‐point scale.

##### Social connection

Perceived social connection was assessed using three items developed for this study. Participants indicated their agreement with statements assessing trust, connectedness and a sense of responsibility towards other Americans (e.g., ‘I can trust most other Americans’; ‘I feel connected to most other Americans’; ‘I feel responsible for the welfare of most other Americans’). Responses were recorded on a scale from 1 (*strongly disagree*) to 7 (*strongly agree*), and items were averaged to form a composite score (*α* = .71).

### Results

As shown in Table 1, perceptions of anomie were not directly associated with either general or specific support for wealth redistribution. However, anomie was positively related to both a desire for drastic societal change and beliefs that the government would misuse redistributed funds. Interestingly, these two variables showed opposing associations with redistribution support: desire for drastic change was positively related to both measures of redistribution support, whereas beliefs about government misuse were negatively related to both measures. Examination of the broader set of measured variables indicated that other candidate mechanisms (i.e., perceived fairness of inequality, general evaluations of inequality, social connection and beliefs that the poor would misuse redistributed funds) showed weaker and less consistent associations with anomie and redistribution support compared to the two focal mediators. On this basis, we conducted a parallel mediation analysis focusing on these two pathways (desire for drastic change and beliefs about government misuse) to assess their potentially contrasting effects.

**TABLE 1:** Correlation and descriptive statistics (Study 1).

	*M*	SD	*N*	1	2	3	4	5	6	7	8	9	10	11
1. Perceptions of anomie	4.30	0.97	573	–										
2. Support for redistribution (gen)	4.81	1.98	573	.02	–									
3. Support for redistribution (specif)	5.40	2.01	572	−.01	.72***	–								
4. Desire for drastic change	4.69	1.45	573	.42***	.39***	.30***	–							
5. Beliefs about gov. misuse	5.11	1.53	573	.55***	−.26***	−.27***	.20***	–						
6. Beliefs about poor misuse	3.19	1.53	573	.11**	−.40***	−.36***	−.16***	.31***	–					
7. Perceived fairness	4.68	1.56	572	.14**	.76***	.65***	.45***	−.16***	−.41***	–				
8. Evaluation of inequality	5.49	1.63	573	.10*	.75***	.68***	.44***	−.16***	−.36***	.77***	–			
9. Social connection	3.95	1.21	573	−.51***	.04	−.04	−.16***	−.35***	−.13**	−.06	−.04	–		
10. Political orientation	3.76	1.82	573	−.01	−.60***	−.60***	−.26***	.27***	.43***	−.55***	−.54***	−.03	–	
11. Subjective social status	5.08	1.67	573	−.24***	−.04	−.06	−.07	−.11**	.08	−.09*	−.09*	.18***	.08*	–

*Note*: *p < .05, **p < .01, ***p < .001.

**FIGURE 1:**
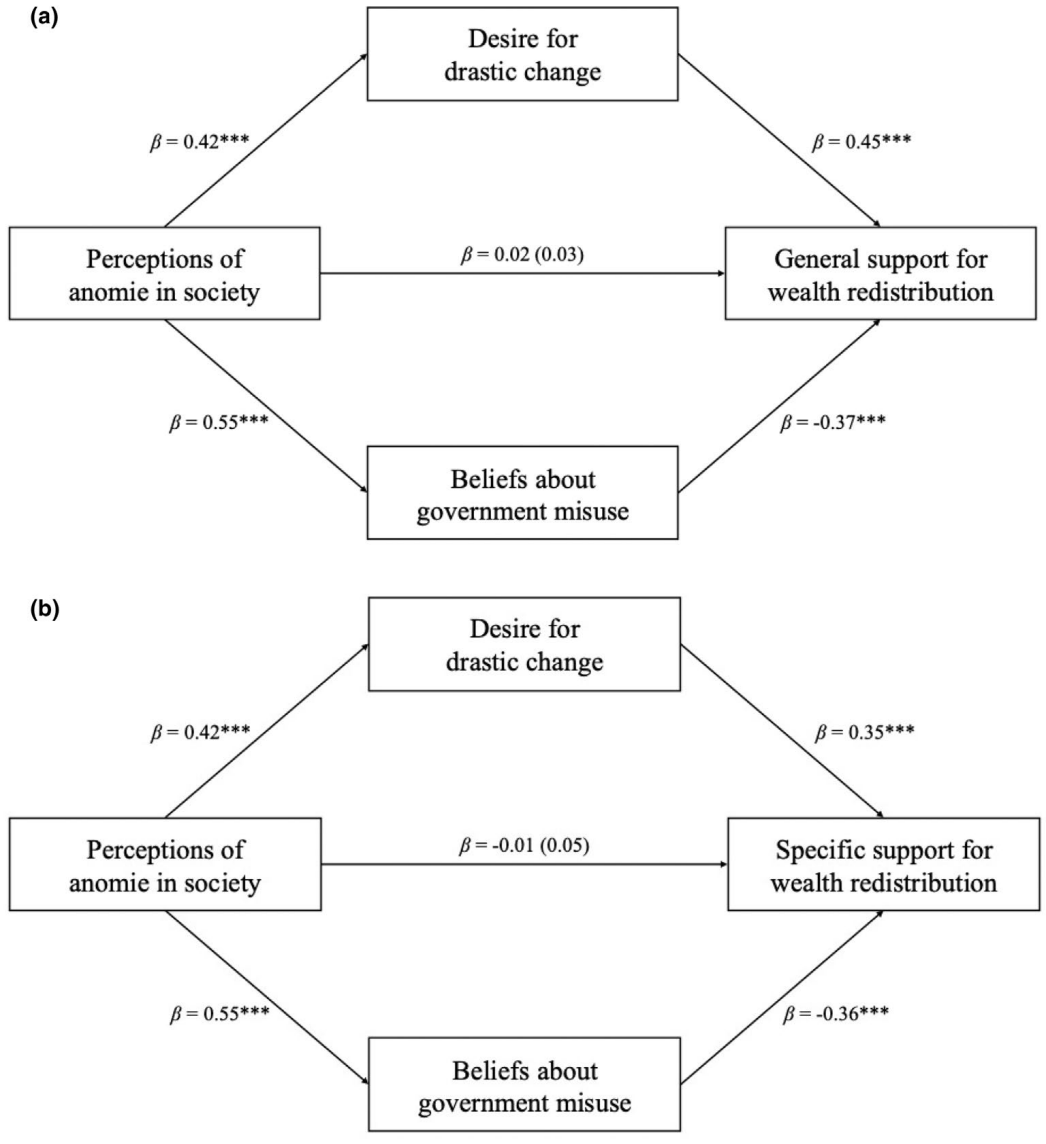
Parallel mediation analyses for Study 1. Panel A depicts the relationship for general support for redistribution, and Panel B depicts the relationship for specific support for redistribution. Standardized coefficients are given. Indirect effects were calculated for each of 5000 bootstrapped samples. The value outside parentheses on the c path is the total effect, and the direct effect is the value inside parentheses. *p < .05, **p < .01, ***p < .001.

See Supplementary Materials S4 for full results for all mediation models (Tables S5–S8). We first tested for parallel mediation for general support for wealth redistribution, followed by specific support for redistribution. A parallel mediation analysis revealed that perceptions of anomie in society are linked to general support for wealth redistribution through two opposing mediators: a desire for drastic change and beliefs about the government's misuse of redistributed wealth (see Figure 1). The total effect of anomie on support for wealth redistribution was not significant, in line with the previous correlation. Importantly, the path from anomie through beliefs about the government's misuse of wealth to support for general wealth redistribution showed a significant negative indirect effect (*β* = −.20, *p* < .001), while the path through a desire for drastic change showed a significant positive indirect effect (*β* = .19, *p* < .001). The direct effect of anomie on general support for wealth redistribution was non‐significant. These findings suggest that while anomie leads to opposing mediatory processes—one reducing and the other increasing support for wealth redistribution—the overall effect cancels out, resulting in no significant direct or total effect. This effect was replicated for specific support for redistribution, further revealing a significant negative indirect effect (*β* = −.20, *p* < .001) for the path via beliefs about government misuse, while the path through a desire for drastic change showed a significant positive indirect effect (*β* = .15, *p* < .001).

We then tested whether the parallel mediation effects held when accounting for political orientation and subjective social status, as these variables are known to affect support for redistribution (Blekesaune, 2013; Brown‐Iannuzzi et al., 2021; Helfer et al., 2024; Kim & Lee, 2018; Noureddine & Gravelle, 2021). For general support for wealth redistribution, the path via beliefs about the government's misuse of wealth showed a significant negative indirect effect (*β* = −.10, *p* < .001), while the path through a desire for drastic change showed a significant positive indirect effect (*β* = .13, *p* < .001). For specific support for wealth redistribution, the path via beliefs about the government's misuse of wealth showed a significant negative indirect effect (*β* = −.09, *p* < .001), while the path through a desire for drastic change showed a significant positive indirect effect (*β* = .09, *p* < .001).^2^

### Discussion

Study 1 revealed no overall association between perceptions of anomie in society and support for wealth redistribution. However, parallel mediation analyses indicated two opposing pathways that appear to neutralize each other. Specifically, perceptions of anomie were associated with greater support for redistribution through an increased desire for drastic change. Conversely, perceptions of anomie were linked to lower support via stronger beliefs that the government would misuse redistributed funds. These effects were consistent across both general and specific measures of wealth redistribution and remained robust after controlling for political orientation and subjective social status. Nevertheless, these results are exploratory, and warrant replication in a novel sample.

## STUDY 2

The aim of Study 2 was to replicate the findings from the previous study.^3^ Here we recruited a sample from the United Kingdom to test the generalizability of our findings beyond the US. This is important as the US is characterized by significant inequality (Chancel et al., 2026), government distrust (Pew Reserach Center, 2025), and polarization (Dimock & Wike, 2021; Edelman, 2023), and these factors may influence perceptions of anomie and support for redistribution. We hypothesized that perceptions of anomie in society will be linked to support for wealth redistribution through two opposing pathways. First, perceived anomie will predict a greater desire for drastic change, which in turn will predict increased support for wealth redistribution. Second, anomie perceptions will predict greater beliefs that the government would misuse redistributed funds, which in turn will predict lower support for wealth redistribution.

### Method

See Supplementary Materials S1 for the full list of items and correlations across items (Table S1).

#### Participants

In total, 453 participants residing in the UK took part in the study. This sample size was based on previous results, where correlations of interest tended to be *r* = .12 or larger. Taking a conservative approach and accounting for budget constraints, a minimum of 428 participants allows us to detect an effect of *r* = .12 with 80% power. To account for exclusions and dropouts, we aimed to collect a final sample of 450 participants. Three participants were removed for failing at least one attention check as preregistered. The final sample was therefore 450 participants. The data was collected from Prolific and, as with the previous study, was representative of the UK in terms of age, sex and political affiliation. The sample was equally split by sex (female = 224; male = 223) and participants were aged between 18 and 79 (*M* = 46.09, SD = 15.80). Participants were centrist on average (*M* = 3.48, SD = 1.49). Participants were also predominantly White (88.7%), tended to perceive themselves as middle class on average (*M* = 5.42; SD = 1.50) and reported low importance of religion on average (*M* = 2.05; SD = 1.77).

#### Measures

We measured additional variables in this study that are not discussed here. These include perceptions of inequality and beliefs that the poor would misuse redistributed wealth and were preregistered as possible exploratory variables.^4^ All measures were otherwise identical to that described in Study 1, including perceptions of anomie (*α* = .87; breakdown in social fabric, *α* = .77; breakdown in leadership, *α* = .86), support for wealth redistribution, need for drastic change (*α* = .84),^5^ and fears of government misuse (*r* = .83).

### Results

As demonstrated in Table 2, there was no significant correlation between perceptions of anomie in society and either measure of support for wealth redistribution. However, perceptions of anomie in society were related to a greater desire for drastic change and a belief that the government would misuse redistributed funds. A desire for drastic change was related to *greater* support for wealth redistribution for both measures. In contrast, a belief that the government would misuse redistributed funds was related to *reduced* support for wealth redistribution for both measures. We therefore conducted a parallel mediation analysis to assess the potential opposing effects.

**TABLE 2:** Correlation and descriptive statistics (Study 2).

	*M*	SD	*N*	1	2	3	4	5	6	7
1. Perceptions of anomie	4.13	0.91	450	–						
2. Support for redistribution (general)	5.40	1.51	450	.07	–					
3. Support for redistribution (specific)	5.83	1.61	450	.02	.64***	–				
4. Desire for drastic change	5.22	1.21	450	.48***	.20***	.15**	–			
5. Beliefs about government misuse	4.80	1.42	450	.62***	−.13**	−.12**	.34***	–		
6. Political orientation	3.48	1.49	450	.18***	−.44***	−.41***	.04	.31***	–	
7. Subjective social status	5.42	1.50	449	−.24***	−.22***	−.18***	−.13**	−.02	.10*	–

*Note*: *p < .05, **p < .01, ***p < .001.

**FIGURE 2:**
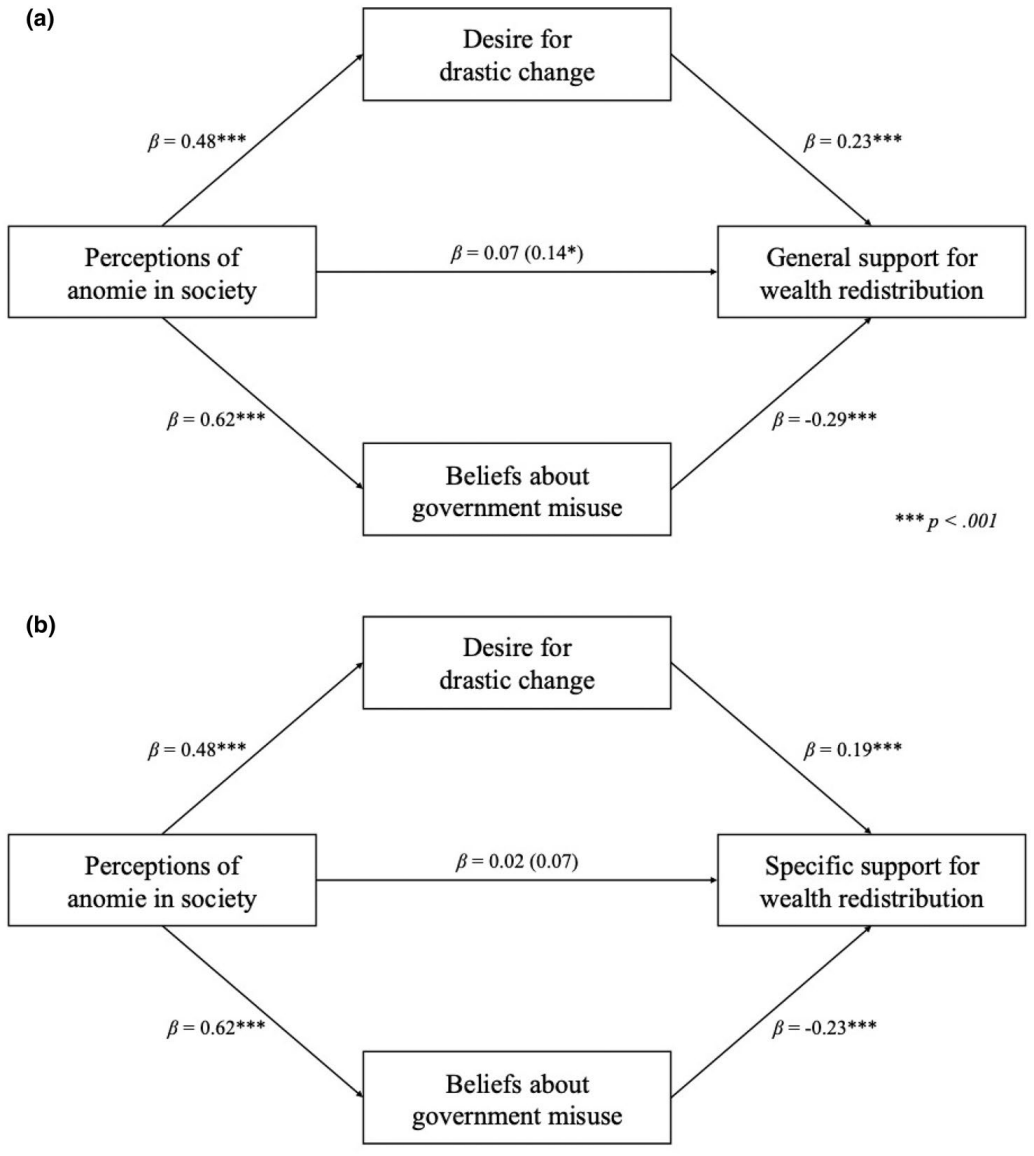
Parallel mediation analyses for Study 2. Panel A depicts the relationship for general support for redistribution, and Panel B depicts the relationship for specific support for redistribution. Standardized coefficients are given. Indirect effects were calculated for each of 5000 bootstrapped samples. The value outside parentheses on the c path is the total effect, and the direct effect is the value inside parentheses. *p < .05, **p < .01, ***p < .001.

See Supplementary Materials S4 for full results for all mediation models (Tables S9–S12). As with Study 1, we first tested for parallel mediation for general support for wealth redistribution, followed by specific support for redistribution. A parallel mediation analysis revealed that perceptions of anomie in society are linked to a general support for wealth redistribution through two opposing mediators: a desire for drastic change and beliefs about the government's misuse of redistributed wealth (see Figure 2). As with Study 1, the total effect of anomie on general support for redistribution was non‐significant. However, significant indirect effects were observed: a negative pathway through beliefs about government misuse (*β* = −.18, *p* < .001) and a positive pathway through desire for drastic change (*β* = .11, *p* < .001). The direct effect of anomie on general support was statistically significant when mediators were included in the model. When examining parallel mediation analysis where specific support for redistribution was the outcome, the total effect was non‐significant. Nevertheless, the model revealed a significant negative indirect effect through beliefs about government misuse (*β* = −.15, *p* < .001) and a significant positive indirect effect through desire for drastic change (*β* = .09, *p* = .001).

We then tested whether the parallel mediation effects held when accounting for political orientation and subjective social status. For general support for wealth redistribution, the path via beliefs about government's misuse of wealth showed a significant negative indirect effect (*β* = −.08, *p* = .018), while the path through a desire for drastic change showed a significant positive indirect effect (*β* = .10, *p* < .001). For specific support for wealth redistribution, the path via beliefs about the government's misuse of wealth was non‐significant (*β* = −.05, *p* = .152), while the path through a desire for drastic change continued to show a significant positive indirect effect (*β* = .08, *p* = .004). The lack of indirect effect via beliefs about the government's misuse of wealth was driven by a non‐significant b path (*β* = −.05, *p* = .146).^6^

### Discussion

As with Study 1, parallel mediation analyses revealed two opposing pathways between perceptions of anomie and support for wealth redistribution. Specifically, perceptions of anomie were associated with greater support for redistribution via a desire for drastic societal change, while simultaneously linked to lower support through stronger beliefs that the government would misuse redistributed funds. However, unlike Study 1, the direct effect of anomie on general support for wealth redistribution was significant, indicating that higher perceptions of anomie were modestly associated with greater support overall after accounting for the mediators. In contrast, the direct effect on specific redistribution remained non‐significant, consistent with the previous findings.

Accounting for political orientation and subjective social status did not affect the results for general support for redistribution. For specific support however, the indirect effect via government misuse was no longer significant for specific redistribution. This may be due to the weaker overall parallel mediation effect in the UK compared to the US. We speculate that, in the US, wealth redistribution is a more politically divisive issue, with strong partisan views often tied to trust in the government, making beliefs about government misuse a central factor in shaping redistribution support. In contrast, the UK's more established welfare state may lead to less polarized views on redistribution, where concerns about government misuse are not as influential in determining support for specific policies.

## STUDY 3

The previous studies found largely consistent evidence that perceptions of anomie in society are linked with both greater support for wealth redistribution (via a greater desire for drastic change) and lower support for wealth redistribution (via greater beliefs the government would misuse redistributed wealth). However, the direction of these pathways is correlational and experimental evidence is needed to understand causality. To that end, we designed the ‘anomie paradigm’ to experimentally test how exposure to a high (vs. low) anomie society affects support for wealth redistribution.

We adapted the established Bimboola paradigm (Salvador Casara et al., 2022; Sánchez‐Rodríguez et al., 2018; Sprong et al., 2019), which presents participants with a fully specified fictional society, and modified it to depict either a high or low anomie context. Recent meta‐analytic work shows that fictional‐society paradigms such as Bimboola are among the most effective methods for experimentally shifting perceptions of complex societal conditions, including economic inequality, supporting the suitability of this framework for manipulating macro‐level perceptions (Sánchez‐Rodríguez et al., 2025). In the present studies, anomie is embedded within a structured fictional environment using quantitative indicators of leadership breakdown and social fabric disintegration. This design allows us to manipulate the core components of anomie in a clear and theory‐consistent way while ensuring coherence between the societal context participants learn about and the redistribution judgements they subsequently make.

We hypothesized that exposure to a high anomie condition (compared to low anomie condition) would cause a greater desire for drastic change, which in turn would predict greater support for wealth redistribution. Moreover, experiencing high anomie (compared to low anomie) would cause greater beliefs that the government would misuse redistributed funds, which in turn would be linked with decreased support for wealth redistribution.

### Method

See Supplementary Materials S1 for the full list of items and correlations across items (Table S2).

#### Participants

In total, 450 participants residing in the United States took part in the study. This sample size was based on results from the previous studies, where effects tended to be small to medium. A minimum of 356 participants allows us to detect an effect of *d* = .35 with 95% power. To account for exclusions and dropouts and to enhance power, we aimed to collect a final sample of 450 participants. Two participants were removed for failing at least one attention check and an additional 18 participants were excluded for failing at least one of three manipulation checks. The final sample was therefore 430 participants. A sensitivity analysis revealed that the final sample can detect an effect size of *d* = 0.24 with 80% power, and *d* = 0.32 with 95% power.

As with the previous studies, data were collected via Prolific Academic using representative sampling procedures, yielding a sample broadly reflective of the U.S. population in terms of age, sex and political affiliation. The sample was equally split by sex (female = 213; male = 212) and participants were aged between 18 and 88 (*M* = 46.45, SD = 16.01). Participants were centrist on average (*M* = 3.60, SD = 1.82) and self‐identified as either Democrat (*n* = 127), Independent (*n* = 182) or Republican (*n* = 116). Participants were predominantly White (74%), followed by Black (10.5%), Asian (6.7%), mixed race (4.9%) or other/prefer not to say (2.6%). Finally, respondents tended to perceive themselves as middle class on average (*M* = 5.12; SD = 1.72) and reported moderate importance of religion (*M* = 3.25; SD = 2.31).

#### Procedure

**FIGURE 3:**
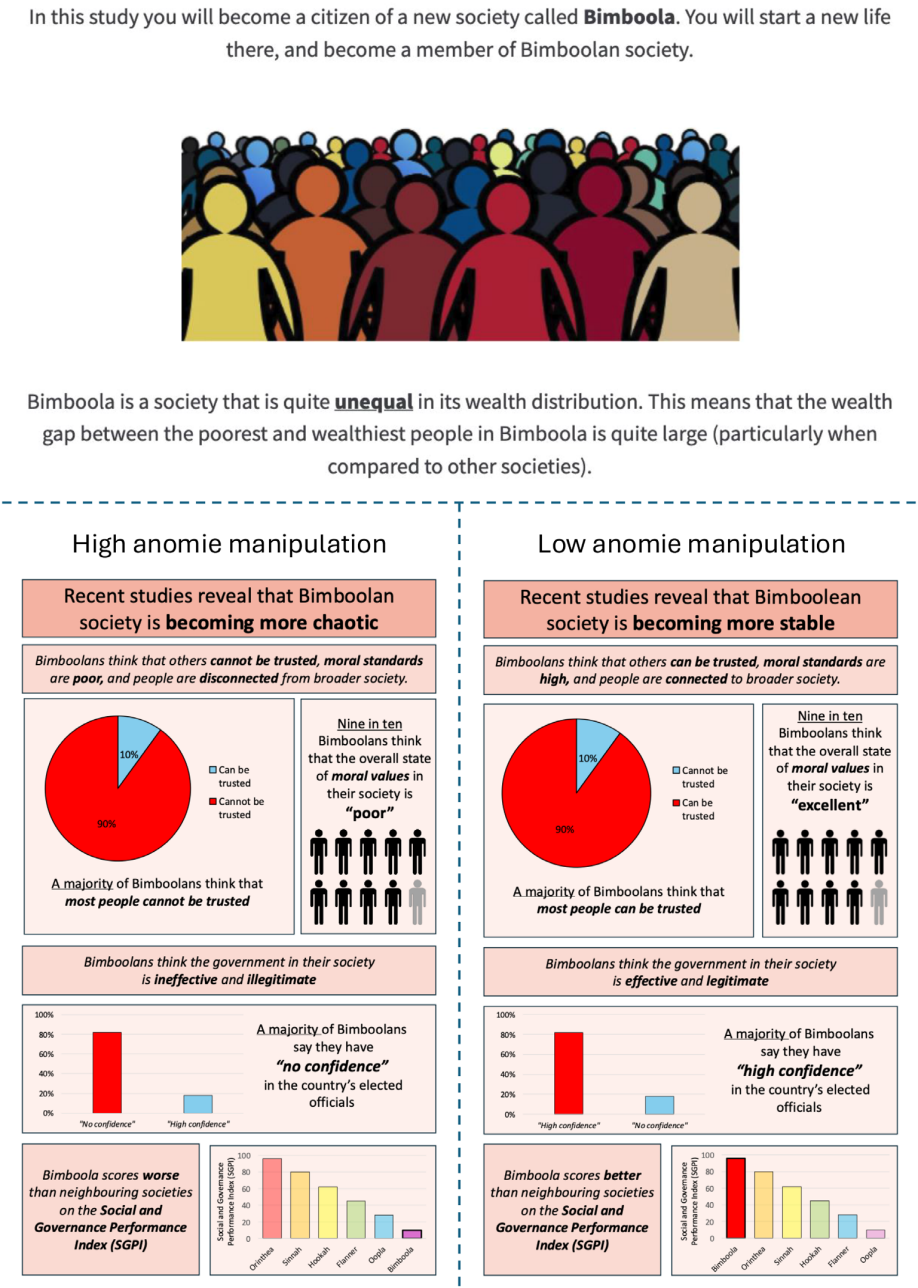
Manipulation for high and low anomie conditions.

After providing consent to participate, participants were told to imagine they were now residents of a fictitious society called Bimboola (see Figure 3). They were first told that Bimboola was an unequal society and then were presented with statistics that either suggested Bimboola was characterized by high anomie (e.g., low trust and moral standards as well as an ineffective and illegitimate government; *n* = 218) or low anomie (e.g., high trust and moral standards as well as an effective and legitimate government; *n* = 212; Teymoori et al., 2016, 2017). Participants then filled out several manipulation checks and control questions, reported their desire for drastic change, beliefs about government misuse of redistributed wealth, support for wealth redistribution and demographic variables.

#### Materials

Participants completed three manipulation checks: whether the information presented suggested Bimboola was stable vs. unstable, whether most people could be trusted, and whether the government could be trusted. To further test manipulation efficacy, participants filled out an adapted anomie scale (Teymoori et al., 2016) framed in the Bimboolan context (e.g., ‘Bimboolan people do not know who they can trust’). Perceptions of inequality were further measured as a control variable with the item, ‘How economically equal or unequal do you think Bimboola is?’ on a scale from 1 (*very equal*) to 7 (*very unequal*). This was to control for the possibility that the anomie manipulations may also alter how unequal participants assume the Bimboolan society is.

Participants additionally reported desire for drastic change^7^ and beliefs about government misuse, adapted to the Bimboolan context (e.g., ‘Drastic changes are needed in Bimboola,’ *α* = .86; ‘The Bimboolan government would misuse that money,’ *r* = .86). They also completed near‐identical redistribution measures as in previous studies, modified for Bimboola (e.g., ‘The government should reduce wealth differences’; ‘Support for a wealth tax on the top 0.1%’).^8^

### Results

We first assessed the effectiveness of the manipulation. A Mann–Whitney U test (conducted due to non‐normality, noting this is a deviation from preregistration) revealed that those in the high anomie condition (*M* = 5.74, SD = 0.64) perceived greater anomie in Bimboola relative to those in the low anomie condition (*M* = 2.67, SD = 0.83; *W* = 46,085, *p* < .001, *r* = 0.86). We then assessed whether the anomie conditions differed in perceptions of inequality. A Mann–Whitney U test (conducted due to non‐normality) revealed that those in the high anomie condition (*M* = 8.03, SD = 2.07) perceived greater inequality in Bimboola relative to those in the low anomie condition (*M* = 6.84, SD = 2.17; *W* = 32,968, *p* < .001, *r* = 0.39). We used perceptions of inequality as a control in all subsequent analyses. We then verified that random allocation to condition was effective and assessed whether the conditions differed in terms of participants' political orientation, religiosity, age and gender. Independent sample *t*‐tests revealed no significant differences between conditions for political orientation (*t*[427.20] = −1.01, *p* = .312), religiosity (*t*[426.84] = −0.07, *p* = .946) and age (*t*[421.02] = 0.08, *p* = .933). Likewise, a Chi‐square test revealed no significant differences between conditions for sex (χ^2^[1] = 0.002, *p* = .962).

An ANCOVA (controlling for perceptions of inequality) found significant differences between conditions in general support for wealth redistribution, such that those in the high anomie condition (*M* = 5.70, SD = 1.57) reported greater general support for wealth redistribution relative to those in the low anomie condition (*M* = 5.04, SD = 1.80; *F*[1, 427] = 16.19, *p* < .001, partial *η* ^2^ = .04). In contrast, an ANCOVA (controlling for perceptions of inequality) found no significant differences between conditions in specific support for wealth redistribution (*F*[1, 427] = 2.89, *p* = .090).

A further ANCOVA (controlling for perceived inequality) revealed a large and significant effect of condition on desire for drastic change. Participants in the high anomie condition (*M* = 6.00, SD = 0.83) reported greater desire for drastic change than those in the low anomie condition (*M* = 3.38, SD = 1.25), *F*(1, 427) = 655.72, *p* < .001, partial *η* ^*2*^ = .61. Finally, an ANCOVA (controlling for perceived inequality) also revealed a large and significant effect of condition on beliefs that the government would misuse redistributed wealth. Participants in the high anomie condition (*M* = 5.67, SD = 1.16) reported greater concerns about government misuse than those in the low anomie condition (*M* = 3.09, SD = 1.46), *F*(1, 427) = 410.59, *p* < .001, partial *η* ^*2*^ = .49.

**FIGURE 4:**
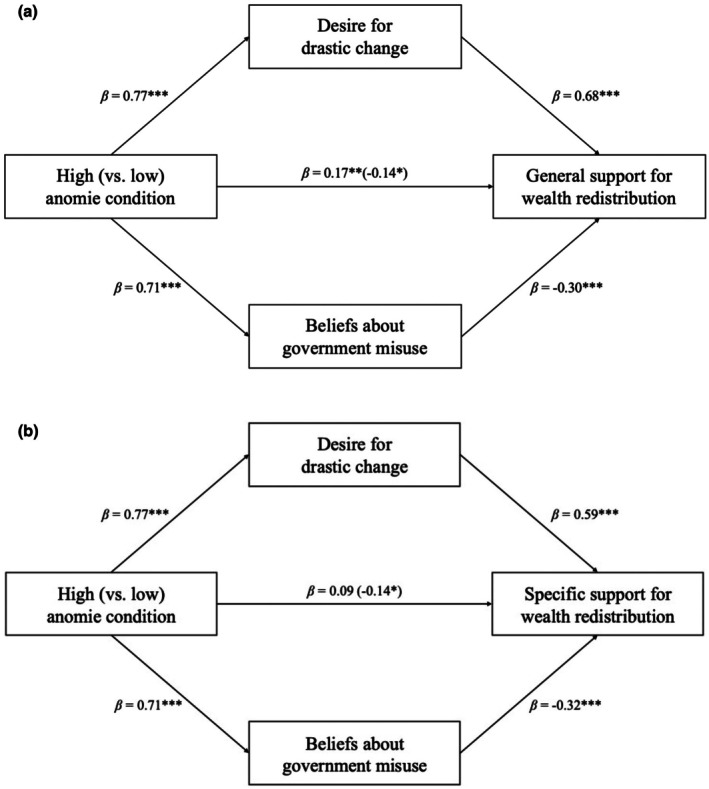
Parallel mediation analyses for Study 3. Panel A depicts the relationship for general support for redistribution and Panel B depicts the relationship for specific support for redistribution. Anomie condition is coded as 1 (low anomie) and 2 (high anomie). Standardized coefficients are given. Indirect effects were calculated for each of 5000 bootstrapped samples. The value outside parentheses on the c path is the total effect, and the direct effect is the value inside parentheses. *p < .05, **p < .01, ***p < .001.

See Supplementary Materials S4 for full results for all mediation models (Tables S13 and S14). After recoding conditions (1 = low anomie, 2 = high anomie), we then conducted parallel mediation analysis for general support for wealth redistribution, followed by specific support for redistribution. Perceptions of inequality were used as a control variable in all analyses. A parallel mediation analysis revealed that perceptions of anomie in society are linked to a general support for wealth redistribution through two opposing mediators: a desire for drastic change and beliefs about the government's misuse of redistributed wealth (see Figure 4).

The total effect of anomie on support for wealth redistribution was significant (*β* = .17, *p* = .001), indicating that those in the high anomie condition had greater support for wealth redistribution in general than those in the low anomie condition. Moreover, the path from anomie through beliefs about the government's misuse of wealth to support for wealth redistribution showed a significant negative indirect effect (*β* = −.21, *p* < .001), while the path through a desire for drastic change showed a significant positive indirect effect (*β* = .52, *p* < .001). The direct effect of anomie on support for wealth redistribution was also significant and negative, *β* = −.14, *p* = .023.

When examining a parallel mediation analysis where specific support for redistribution was the outcome, the total effect was non‐significant. Importantly, the model for specific support for wealth redistribution revealed a significant negative indirect effect (*β* = −.23, *p* < .001) for the path via beliefs about government misuse, while the path through a desire for drastic change showed a significant positive indirect effect (*β* = .46, *p* < .001). The direct effect was significant and negative, *β* = −.14, *p* = .039.

### Discussion

Study 3 aimed to confirm the directionality of the relationships found in Studies 1 and 2. In line with our hypotheses, parallel mediation analyses revealed two opposing pathways linking anomie to support for wealth redistribution. Specifically, the high anomie condition (compared to low anomie) led to greater support for redistribution through an increased desire for drastic change. At the same time, heightened anomie was associated with lower support for redistribution, via stronger beliefs that the government would misuse the redistributed funds. Unlike Study 1 and 2, the total effect of anomie on support for general wealth redistribution was significant, indicating that exposure to a high versus low anomie society caused greater support overall. Consistent with the previous findings, the total effect on specific redistribution remained non‐significant.

## GENERAL DISCUSSION

High economic inequality exacerbates a range of societal ills, and addressing high levels of economic inequality may be crucial for alleviating these broader issues in society (Piketty, 2020; Wilkinson & Pickett, 2009; Zucman, 2014). This underscores the need to understand barriers to public support for wealth redistribution, a key policy tool for reducing extreme capital concentration. Here, we examine how perceptions of societal breakdown, or anomie, shape attitudes towards wealth redistribution. Our first two studies investigated whether perceptions of anomie in society are linked to support for redistribution through two opposing mechanisms: a desire for drastic change and concerns about government misuse of redistributed resources. In our third study, we experimentally tested how being exposed to a society characterized by high (versus low) anomie affects support for wealth redistribution.

In Studies 1 and 2, perceptions of anomie had no overall relationship with support for wealth redistribution. Instead, perceptions of anomie were related to support for wealth redistribution via two opposing mechanisms. On one hand, individuals who perceived greater anomie expressed a stronger desire for drastic societal change, which in turn was related to *increased* support for redistribution. On the other hand, heightened perceptions of anomie were also related to greater concerns about government misuse of redistributed wealth, ultimately linking to *reduced* support for redistribution. These findings suggest that perceptions of anomie can give rise to complex and potentially offsetting responses to wealth redistribution, as the same perceptions simultaneously activate motivations for societal change as well as concerns about how redistributed resources would be managed.

It is worth noting, however, that in the UK sample, but not the US sample, the indirect effect of perceptions of anomie on support for a specific wealth redistribution policy (wealth tax on the 0.1%), via concerns about government misuse, became non‐significant once controlling for political orientation and subjective social status. These control variables, however, did not alter the relationships observed for *general* support for wealth redistribution. This may reflect the weaker mediation effect observed in the UK compared to the US, where beliefs about government misuse appear to play a more central role. However, this finding is exploratory, and future research is needed to clarify how political orientation and cultural context shape these relationships.

An important question is whether the two mediators identified in Studies 1 and 2 represent distinct psychological responses to perceived anomie, or whether they instead reflect overlapping reactions tied to different dimensions of anomie perceptions. In particular, one might argue that a desire for drastic change primarily stems from perceptions of breakdown in the social fabric, whereas concerns about government misuse simply reflect perceived breakdown in leadership. However, our supplemental analyses do not support this interpretation. When the two anomie dimensions were examined simultaneously, both independently predicted desire for drastic change and concerns about misuse in patterns consistent with our theoretical model, with only limited and sample‐specific attenuation. This suggests that the two mediators capture meaningfully distinct motivational responses to perceptions of anomie more broadly.

Study 3 then confirmed the directionality of the mechanisms by manipulating the degree of anomie in a fictitious society. Here, the high anomie condition was related to greater support for wealth redistribution relative to the low anomie condition, and this diverged from the previous studies where no overall effect was found. Importantly, participants exposed to the high (compared to low) anomie condition exhibited a stronger desire for drastic societal change, in turn reporting increased support for wealth redistribution. Simultaneously, the high (compared to low) anomie condition heightened concerns about government misuse of redistributed wealth, which was then linked with reduced support for redistribution.

This work contributes to a theoretical understanding of how perceptions of anomie in society can affect human psychology (Teymoori et al., 2017). Rather than producing a single uniform response, perceptions of anomie may activate multiple psychological reactions. These include both an increased motivation for drastic societal change (Kirkland et al., 2024; Sprong et al., 2019) and feelings of hopelessness (Norasakkunkit & Uchida, 2011; Teymoori et al., 2017; Thorlindsson, 2004). Together, these reactions reflect the dual psychological impact of anomie, whereby the collapse of societal norms and the perceived illegitimacy of governing institutions can simultaneously strengthen motivations for radical change while also increasing concerns about how collective resources would be managed. These processes may differ in their temporal focus, which helps explain how they can operate in parallel. A desire for drastic change is often future‐oriented and reflects aspirations for long term transformation, while concerns about government misuse are grounded in more immediate mistrust of current institutions. Both evaluations may coexist within the same individual and contribute to ambivalence towards redistribution. Future research could examine this temporal asymmetry more directly by manipulating the perceived time horizon of redistribution and testing whether future‐oriented framings increase the relative salience of desire for drastic change, while present‐focused framings heighten concerns about government misuse, without necessarily eliminating the other pathway.

Taken together, these processes highlight how perceptions of anomie may shape political attitudes through multiple, co‐occurring psychological mechanisms. This perspective complements a substantial body of research examining the factors that influence support for wealth redistribution, which has largely focused on individual‐level characteristics such as ideology, self‐interest, and perceptions of inequality (Armingeon & Weisstanner, 2022; Brown‐Iannuzzi et al., 2021; Knell & Stix, 2020; Norton & Ariely, 2011; Walker et al., 2021). However, comparatively little work has focused on the broader socio‐structural conditions that shape this support. Here, we demonstrate that the perception of societal breakdown is a critical and complex factor that should be considered.

From a practical perspective, the present findings suggest that the relationship between perceived societal anomie and support for wealth redistribution is unlikely to be straightforward. When anomie is salient, perceptions of societal breakdown can give rise to opposing psychological responses, including motivations for structural change and concerns about institutional competence, which may be difficult to reconcile in practice. As a result, the net association between anomie and redistribution support will depend on which of these responses is more prominent in a given context. In contexts where aspirations for large‐scale change dominate, perceptions of anomie may be associated with greater openness to redistributive policies, whereas when concerns about misuse or ineffective implementation are more salient, the same perceptions may dampen support. More broadly, these findings suggest that reductions in perceived societal anomie, and the accompanying restoration of confidence in institutions, may help create conditions under which support for redistribution is more likely to emerge.

The current research offers several notable strengths. We replicated correlational findings across two distinct populations and confirmed the causal relationship through experimental evidence. Using two outcome measures (a general desire to reduce the wealth gap and a specific preference for a wealth tax) allows us to test if the influence of perceptions of anomie is robust across both stable and more flexible attitudes. By including an elastic, policy‐specific measure (Kuziemko et al., 2015), we can assess whether anomie's impact extends to concrete policy support, which is often more susceptible to contextual shifts.

Nonetheless, there are limitations that future research should address. Although the experiment successfully manipulated perceptions of anomie, it did so within a fictitious context, which limits the generalizability of the findings. To strengthen the external validity of these results, future research should explore whether manipulating perceptions of anomie about the real‐world influences *actual* support for redistribution. Additionally, future research should explore behavioural outcomes, such as voting or petition signing. Moreover, although we tested our hypotheses in two samples, the United States and the United Kingdom, these are W.E.I.R.D. populations (Henrich et al., 2010). As such, the generalizability of our findings to more culturally diverse or non‐Western contexts remains uncertain.

An additional limitation of the present studies concerns how beliefs about government misuse were measured. Participants were asked to evaluate statements about whether the government could be trusted to ‘*use those funds correctly*’ in a scenario explicitly framed around redistribution (i.e., ‘*Imagine the government started taxing the rich with the intention of giving that money to the poor*’). As a result, some participants may have endorsed these items not because they expected the government to misuse funds, but because they were generally opposed to redistribution itself. Future research could reduce this ambiguity by assessing concerns about government mismanagement in contexts that are not directly tied to redistributive policies.

Moreover, support for redistribution was assessed using single‐item measures, which may limit reliability. Future research may wish to employ multi‐item validated scales to more fully capture redistribution attitudes. Finally, it remains unclear whether concerns about government misuse under perceived anomie reflect reduced support for redistribution as implemented by *current* governing institutions or a more enduring shift in general redistributive preferences. Future research should disentangle these possibilities.

Economic inequality continues to grow in many places around the world (Piketty, 2020; Schifferes, 2020), underscoring the need to understand the factors that promote or inhibit public support for wealth redistribution. Here, we demonstrated that perceptions of anomie lead to complex and nuanced effects on support for wealth redistribution. On one hand, feelings of societal breakdown can fuel a desire for drastic change, fostering greater support for redistributive policies. On the other hand, these same perceptions can heighten concerns about how effectively redistributive policies would be implemented, thereby decreasing support for wealth redistribution. These findings highlight the complex ways in which perceptions of anomie shape public opinion on support for wealth redistribution. As society grapples with unprecedented division and decay, understanding the psychological impact of these perceptions is critical to navigating the many challenges ahead.

## AUTHOR CONTRIBUTIONS

**Kelly Kirkland:** Conceptualization; investigation; funding acquisition; writing – original draft; writing – review and editing; visualization; methodology; formal analysis; project administration; resources; data curation. **Christoph Klebl:** Conceptualization; methodology; writing – review and editing. **Christian T. Elbæk:** Writing – review and editing; conceptualization. **Jolanda Jetten:** Supervision; conceptualization; methodology; investigation; writing – review and editing.

## ACKNOWLEDGEMENTS

Open access publishing facilitated by The University of Queensland, as part of the Wiley ‐ The University of Queensland agreement via the Council of Australasian University Librarians.

## FUNDING INFORMATION

Christian T. Elbæk was supported by grant AUFF‐E‐2023‐9‐64 from Aarhus University Research Foundation (AUFF). Jolanda Jetten was supported by an Australian Laureate Fellowships (FL180100094).

## CONFLICT OF INTEREST STATEMENT

The authors declare no conflicts of interest.

## Supporting information


Data S1.

## Data Availability

All studies were preregistered on the Open Science Framework prior to data collection (Study 1: https://osf.io/4t5ke/?view_only=bf0eb167efbc42619edcba0f8046f7f1; Study 2: https://osf.io/745zr/?view_only=8f2cd91c82364a82a5c58e2eaa9bbe1e; Study 3: https://osf.io/pg7jr/?view_only=247951955bdb4eb9bef2c36f71d30344). Materials, data and analysis code can be accessed via the following link: https://osf.io/ayjzn/?view_only=5c14c0df2adb4ad0b1c709677c43b5f8.

## Footnotes

^1^
See Supplementary Materials S3 for results with full scale for Study 1 and 2 (Tables S3 and S4; Figures S2 and S3).

^2^
See Supplementary Materials S5 for results per anomie subscale (Tables S15 and S16; Figures S4 and S5).

^3^
We conducted another study with the same aims but excluded it due to a wording error in a key item. Supplementary Materials S6 provides details and results (Table S19; Figure S8). The error did not meaningfully affect outcomes. The study reported here in the manuscript was from a novel sample with the corrected materials.

^4^
See Supplementary Materials S1 for additional information.

^5^
As in Study 1, the reverse‐coded item (‘Quick fixes are possible and sufficient’) was problematic (α = .67) and removed.

^6^
See Supplementary Materials S5 for results per anomie subscales (Tables S17 and S18; Figures S6 and S7).

^7^
All four items were averaged due to sufficient reliability.

^8^
We also measured beliefs that the poor would misuse redistributed wealth, preregistered as exploratory. Details are in Supplementary Materials S1.
