# Supplementary material for: Perceptions of anomie in society shape support for wealth redistribution
Source: Br J Soc Psychol. 2026 Mar 29;65(2):e70067. doi: 10.1111/bjso.70067 (PMC13033917; doi:10.1111/bjso.70067)
Supplement: Supplementary file 1 — Data S1. [file BJSO-65-0-s001.docx]

**Supplementary Materials**

Table of Contents

[Supplementary Materials 1 – Full List of Items Across Studies 2](#_Toc216879080)

[Supplementary Materials 2 – Anomie Manipulation from Study 1 8](#_Toc216879081)

[Supplementary Materials 3 – Results with Full Drastic Change Scale for Study 1 and 2 10](#_Toc216879082)

[Supplementary Materials 4 – Full Mediation Output for all Studies 12](#_Toc216879083)

[Supplementary Materials 5 – Results for all Studies by Anomie Subscale 22](#_Toc216879084)

[Supplementary Materials 6 – Results for Unreported Study with Wording Error 27](#_Toc216879085)

[References 29](#_Toc216879086)

# Supplementary Materials 1 – Full List of Items Across Studies

**Study 1**

***Manipulation checks^[[1]](#footnote-1)^***

- "The information I was presented with suggests that American society is:"

*1 = Very stable, 2 = Very unstable*

- "The information I was presented with suggests that most people:"

*1 = Can be trusted, 2 = Cannot be trusted*

- *“*The information I was presented with suggests that the government:"

*1 = Can be trusted, 2 = Cannot be trusted*

***Anomie (Teymoori et al., 2016, 2017)***

- In the social fabric
  - People think that there are no clear moral standards to follow
  - Everyone thinks of himself/herself and does not help others in need
  - Most people think that if something works, it doesn’t really matter whether it is right or wrong
  - People do not know who they can trust and rely on
  - Most of the people think that honesty doesn’t work all the time; dishonesty is sometimes a better approach to get
  - People are cooperative
- In leadership
  - The government works towards the welfare of people
  - The government is legitimate
  - The government uses its power legitimately
  - Politicians don’t care about the problems of average person
  - The government laws and policies are effective
  - Some laws are not fair

*Strongly disagree (1) to strongly agree (7)*

***Support for wealth redistribution (general; adapted from European Social Survey, 2020)***

- The United States government should take measures to reduce differences in wealth levels

*Strongly disagree (1) to strongly agree (7)*

***Support for wealth redistribution (specific; Elbæk et al., 2024)***

- How much would you be in favor of a wealth tax on the top 0.1%?

*Strongly oppose (1) to strongly favour (7)*

***Need for drastic change (Klebl & Jetten, 2024)***

- Drastic changes are needed
- Quick fixes are possible and sufficient
- Don’t patch it up – shake it up
- We cannot go on like this and therefore have to change the system

*Not at all (1) to very much so (7)*

***Fear of wealth misuse by government (created for this study)***

Imagine the American government started taxing the rich with the intention of giving that money to the poor. How much do you agree with the following statements:

- We can’t trust the government to use those funds correctly
- The government would misuse that money

*Strongly disagree (1) to strongly agree (7)*

***Fear of wealth misuse by poor (created for this study)***

Imagine the American government started taxing the rich with the intention of giving that money to the poor. How much do you agree with the following statements:

- We can’t trust poor people to use those funds correctly
- Poor people would misuse that money

*Strongly disagree (1) to strongly agree (7)*

***Explicit Fairness (Schmalor & Heine, 2021)***

- It is extremely unfair if the overall amount of economic inequality is very high
- It is not fair at all if there are large differences in income between the rich and the poor
- It is immoral if your income is dependent on where you grew up
- It is extremely unjust if children of affluent parents get a better education

*Strongly disagree (1) to strongly agree (7)*

***Implicit Fairness (adapted from ISSP; Elbæk et al., 2024)***

- Differences in wealth in the United States are too large.

*Strongly disagree (1) to strongly agree (7)*

***Social connection (created for this study)***

- I can trust most other Americans
- I feel connected to most other Americans
- I feel responsible for the welfare of most other Americans

*Strongly disagree (1) to strongly agree (7)*

***Age***

- Age (in years): ______________

***Gender***

- Gender: ______________

***Economic conservatism***

- Please indicate your political beliefs from left/liberal to right/conservative on issues of the economy (e.g., social welfare, government spending, tax cuts):

*Left/liberal (1) to right/conservative (7)*

***Social conservatism***

- Please indicate your political beliefs from left/liberal to right/conservative on social issues (e.g., immigration, homosexual marriage, abortion):

*Left/liberal (1) to right/conservative (7)*

***MacArthur Scale of Subjective Social Status***

- Think of this ladder as representing where people stand in the US. At the top of the ladder are the people who have the most money, most education, and most respected jobs. At the bottom are the people who have the least money, least education, and least respected jobs or no job. The higher up you are on this ladder, the closer you are to the people at the very top, and the lower you are, the closer you are to the people at the very bottom. Where would you place yourself on this ladder? Please select a number corresponding to the rung where you think you stand at this time in your life, relative to other people in the US.


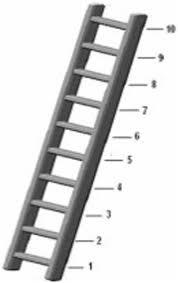


*Least money, job prestige and education (1) to most money, job prestige and education (10)*

***Religiosity***

- How important is religion in your daily life? If you do not follow a religion, please select: 1 (not at all important).

*Not at all important (1) to extremely important (7)*

**Study 2**

Anomie, support for wealth redistribution (general and specific), desire for drastic change, fear of wealth misuse by government, fear of wealth misuse by poor (not analysed; *M* = 3.34, *SD* = 1.62), and demographics were measured identical to Study 1. However, where appropriate, these referenced the United Kingdom rather than the United States. We further included the following measures:

***Implicit perceptions of inequality (Kirkland et al., 2022, 2024; Sprong et al., 2019)***

- We would like you to think of 100 citizens of the United Kingdom. How many of these 100 people would in your view be classified as “very poor”, “poor”, “average in wealth”, “wealthy”, and “very wealthy”? Please write the number of people in each box. Please make sure the estimates add up to 100 people.
  - Very poor:
  - Poor:
  - Average in wealth:
  - Wealthy:
  - Very wealthy:

*See previous research for calculation to achieve a quasi-Gini coefficient.*

***Explicit perceptions of inequality (Kirkland et al., 2022)***

- We would like you to think of the poorest and the wealthiest people in the United Kingdom. Overall, how large is the wealth gap between the poorest and the wealthiest people in the United Kingdom?

*Very small (1) to very large (7)*

***Table S1.***

*Correlation table (Study 2) for Relationship between Fear of Wealth Misuse by the Poor and Other Variables*

|  | 1 |
| --- | --- |
| 1. Fears of wealth misuse (poor) | — |
| 2. Perceptions of anomie | .12** |
| 3. Support for redistribution (general) | -.44*** |
| 4. Support for redistribution (specific) | -.40*** |
| 5. Desire for drastic change | -.05 |
| 6. Beliefs about government misuse | .25*** |

*Note*. * *p* < .05 ** *p* < .01 *** *p* < .001

**Study 3**

Below are items included in the study, adapted from the previous two studies to be in reference to the Bimboolan context. Demographic variables were measured in an identical manner to the previous two studies.

***Manipulation checks***

- "The information I was presented with suggests that Bimboolan society is:"

*1 = Very stable, 2 = Very unstable*

- "The information I was presented with suggests that most people in Bimboola:"

*1 = Can be trusted, 2 = Cannot be trusted*

- *“*The information I was presented with suggests that the government in Bimboola:"

*1 = Can be trusted, 2 = Cannot be trusted*

***Inequality check***

- How economically equal or unequal do you think Bimboola is?

*Very equal (1) to very unequal (7)*

***Anomie***

- In the social fabric
  - People in Bimboola think that there are no clear moral standards to follow
  - Everyone in Bimboola thinks of himself/herself and does not help others in need
  - Most people in Bimboola think that if something works, it doesn’t really matter whether it is right or wrong
  - Bimboolan people do not know who they can trust and rely on
  - Most Bimboolan people think that honesty doesn’t work all the time; dishonesty is sometimes a better approach to get ahead
  - The people of Bimboola are cooperative
- In leadership
  - The Bimboolan government works towards the welfare of people
  - The Bimboolan government is legitimate
  - The Bimboolan government uses its power legitimately
  - Politicians in Bimboola don’t care about the problems of average person
  - The government laws and policies are effective in Bimboola
  - Some laws in Bimboola are not fair

*Strongly disagree (1) to strongly agree (7)*

***Support for wealth redistribution (general)***

- The Bimboolan government should take measures to reduce differences in wealth levels

*Strongly disagree (1) to strongly agree (7)*

***Support for wealth redistribution (specific)***

- How much would you be in favor of a wealth tax on the top 0.1% of Bimboola?

*Strongly oppose (1) to strongly favour (7)*

***Need for drastic change***

- Drastic changes are needed in Bimboola
- Quick fixes are possible and sufficient in Bimboola
- In Bimboola, we can't just patch it up – we need to shake it up
- We cannot go on like this and therefore have to change the system in Bimboola

*Not at all (1) to very much so (7)*

***Fear of wealth misuse by government***

The Bimboolan government is considering a new policy, where they propose to tax the rich and give that money to the poor. How much do you agree with the following statements:

- We can’t trust the Bimboolan government to use those funds correctly
- The Bimboolan government would misuse that money

*Strongly disagree (1) to strongly agree (7)*

***Fear of wealth misuse by poor *Not analysed***

The Bimboolan government is considering a new policy, where they propose to tax the rich and give that money to the poor. How much do you agree with the following statements:

- We can’t trust the poor people of Bimboola to use those funds correctly
- The poor people of Bimboola would misuse that money

*Strongly disagree (1) to strongly agree (7)*

**Results for Condition Effects on Wealth Misuse by Poor**

An ANCOVA was conducted to examine whether the anomie manipulation influenced beliefs that the poor would misuse redistributed wealth, controlling for perceived inequality. There was a significant main effect of condition, *F*(1, 427) = 46.42, *p* < .001, partial η² = .10, such that participants in the high anomie condition reported stronger beliefs that the poor would misuse redistributed funds (*M* = 3.51, *SD* = 1.75) than those in the low anomie condition (*M* = 2.49, *SD* = 1.31).

***Table S2.***

*Correlation table (Study 3) for Relationship between Fear of Wealth Misuse by the Poor and Other Variables*

|  | 1 |
| --- | --- |
| 1. Fears of wealth misuse (poor) | — |
| 2. Perceptions of anomie | .33*** |
| 3. Support for redistribution (general) | -.25*** |
| 4. Support for redistribution (specific) | -.34*** |
| 5. Desire for drastic change | .20*** |
| 6. Beliefs about government misuse | .50*** |

*Note*. * *p* < .05 ** *p* < .01 *** *p* < .001

# Supplementary Materials 2 – Anomie Manipulation from Study 1

Study 1 was initially designed to manipulate actual perceptions of anomie in society, by gathering statistics that suggested American society was characterised by high anomie, low anomie, or an empty control condition. However, this manipulation failed, and details are provided below on basic statistics. The manipulation likely failed for several reasons, including limitations using real statistics^[[2]](#footnote-2)^ (affecting the ability to find genuine statistics that suggest the US is characterised by low anomie) and general difficulty in shifting real-world beliefs and attitudes. Due to indications the manipulation failed, we only reported correlational statistics in the main manuscript as exploratory findings. Outcome measures reported below are described in Supplementary Materials 1 for Study 1.

Participants were assigned to either a high anomie (*n* = 191), low anomie (*n* = 184) or empty control condition (*n* = 198; see Figure S1)^[[3]](#footnote-3)^. A one-way ANOVA revealed significant differences between conditions in perceptions of anomie, *F*(2,570) = 11.40, *p* < .001. Tukey’s HSD post-hoc tests showed that perceptions of anomie were significantly higher in the High Anomie condition (*M* = 4.56, *SD* = 0.93) than in the Control condition (*M* = 4.24, *SD* = 0.91), *p* = .004, *d* = 0.34, and significantly lower in the Low Anomie condition (*M* = 4.10, *SD* = 1.02) than in the High Anomie condition, *p* < .001, *d* = 0.47. However, there was no significant difference between the Low Anomie and Control conditions, *p* = .301.This broadly suggests that the High Anomie manipulation effectively enhanced perceptions of anomie with a small to medium effect, whereas the Low Anomie manipulation failed to effectively reduce perceptions of anomie.

***Figure S1.*** Manipulation of Anomie (High and Low Anomie Conditions)


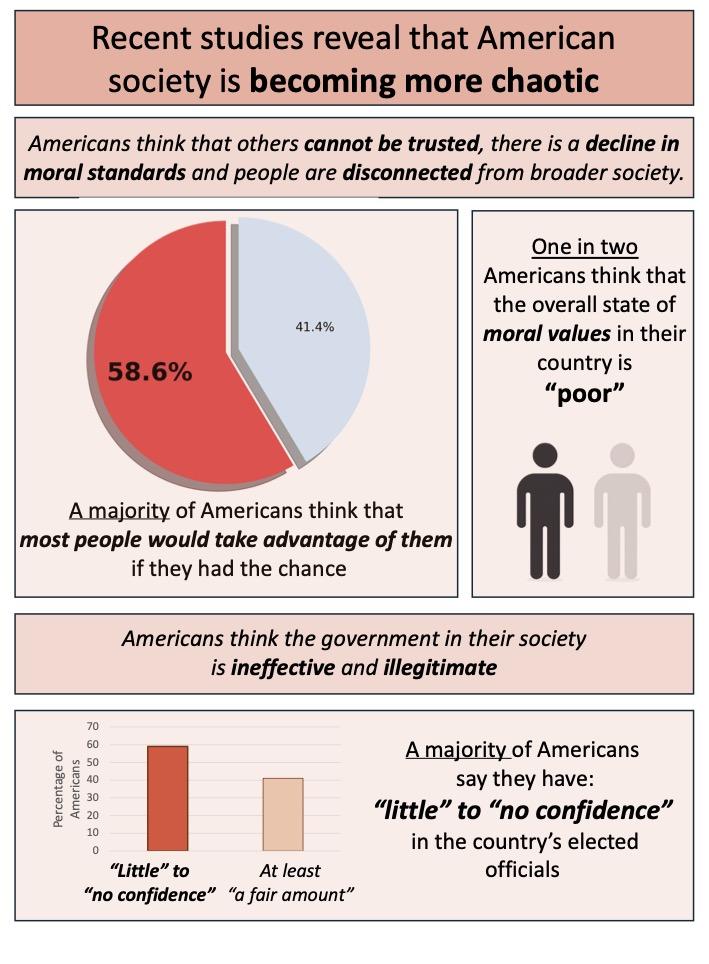

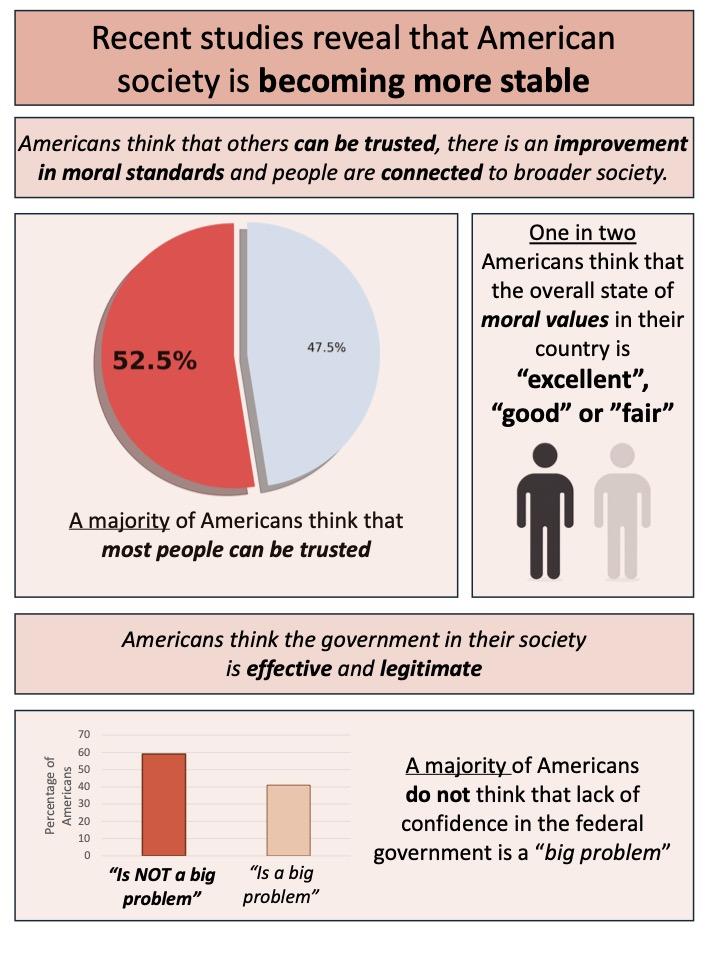


A one-way ANOVA was also conducted to examine the effect of Condition on general support for wealth redistribution, which revealed no significant differences, *F*(2, 570) = 0.06, *p* = .938. Similarly, there were no significant differences between conditions in specific support for wealth redistribution, *F*(2, 569) = 0.13, *p* = .878. A one-way ANOVA was conducted to examine the effect of Condition on perceptions of government misuse of wealth, revealing no significant differences, *F*(2, 570) = 0.81, *p* = .448. Likewise, there were no significant differences between conditions in support for drastic change, *F*(2, 570) = 0.31, *p* = .733.

# Supplementary Materials 3 – Results with Full Drastic Change Scale for Study 1 and 2

**Study 1**

***Table S3.***

*Correlation table (Study 1)*

|  | 1 | 2 | 3 | 4 | 5 |
| --- | --- | --- | --- | --- | --- |
| 1. Perceptions of anomie | – |  |  |  |  |
| 2. Support for redistribution (general) | .02 | – |  |  |  |
| 3. Support for redistribution (specific) | -.01 | .72*** | – |  |  |
| 4. Desire for drastic change | .41*** | .34*** | .26*** | – |  |
| 5. Beliefs about government misuse | .55*** | -.26*** | -.27*** | .23*** | – |

*Note*. * *p* < .05 ** *p* < .01 *** *p* < .001

***Figure S2.*** Parallel Mediation Analyses for Study 1


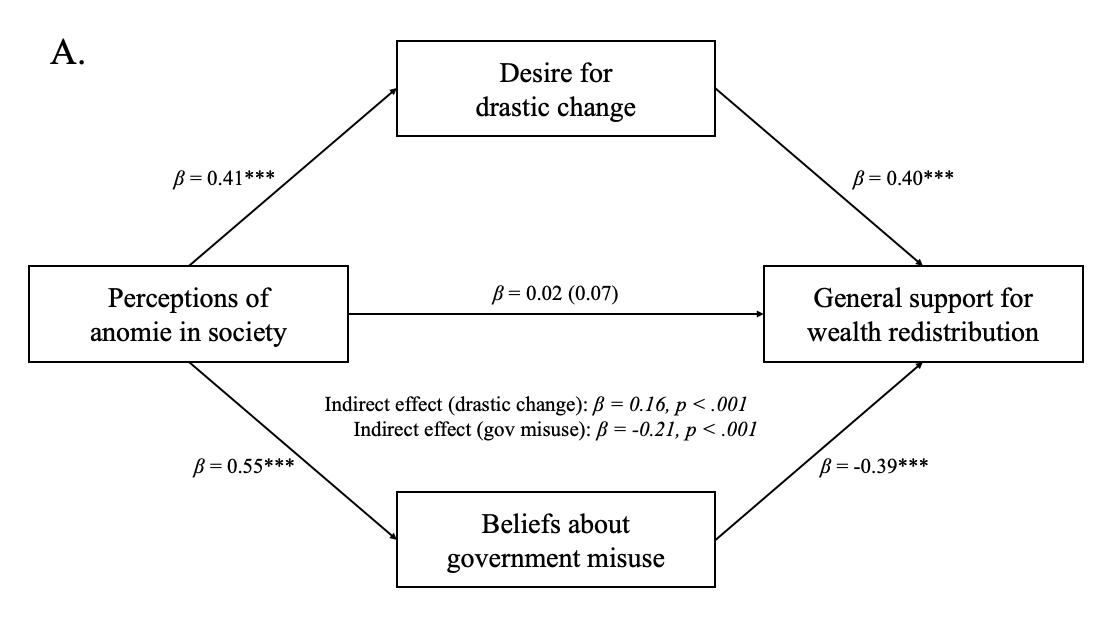

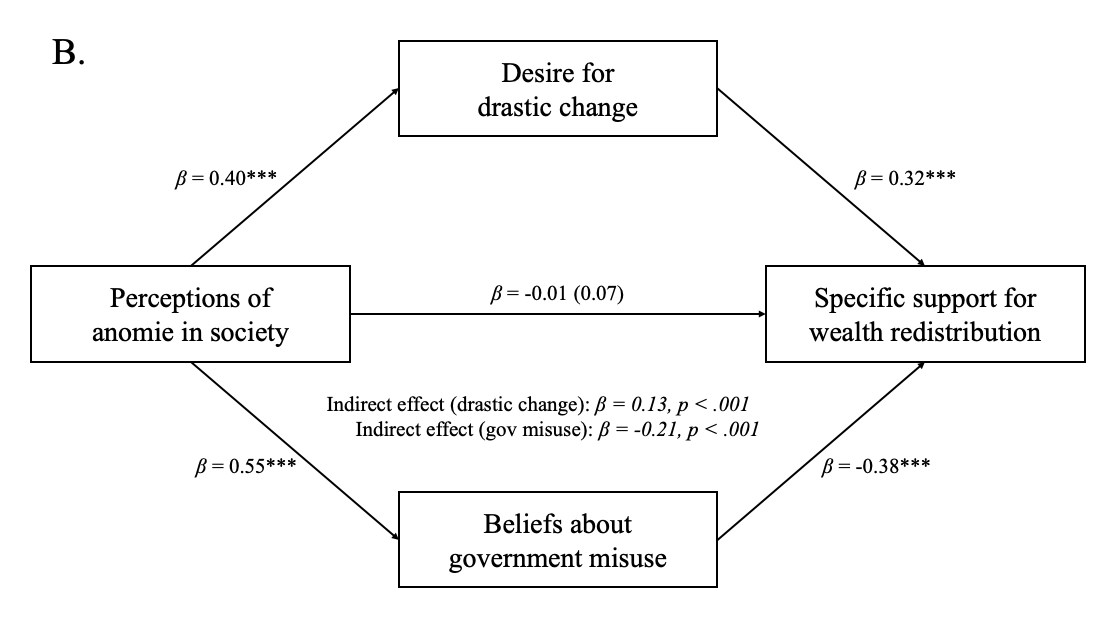


*Note:* Panel A depicts the relationship for general support for redistribution and Panel B depicts the relationship for specific support for redistribution. Standardized coefficients are given. Indirect effects were calculated for each of 5000 bootstrapped samples. The value outside parentheses on the c path is the total effect, and the direct effect is the value inside parentheses.

* *p* < .05 ** *p* < .01 *** *p* < .001

**Study 2**

***Table S4.***

*Correlation table (Study 2)*

|  | 1 | 2 | 3 | 4 | 5 |
| --- | --- | --- | --- | --- | --- |
| 1. Perceptions of anomie | – |  |  |  |  |
| 2. Support for redistribution (general) | .07 | – |  |  |  |
| 3. Support for redistribution (specific) | .02 | .64*** | – |  |  |
| 4. Desire for drastic change | .47*** | .19*** | .14** | – |  |
| 5. Beliefs about government misuse | .62*** | -.13** | -.12** | .35*** | – |

*Note*. * *p* < .05 ** *p* < .01 *** *p* < .001

***Figure S3.*** Parallel Mediation Analyses for Study 2


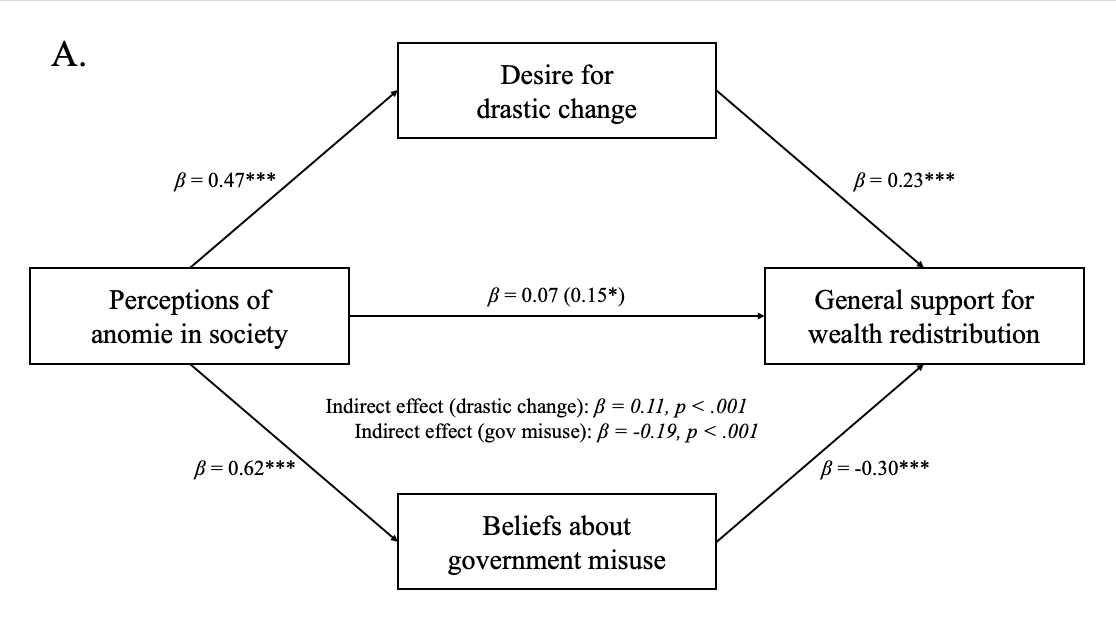

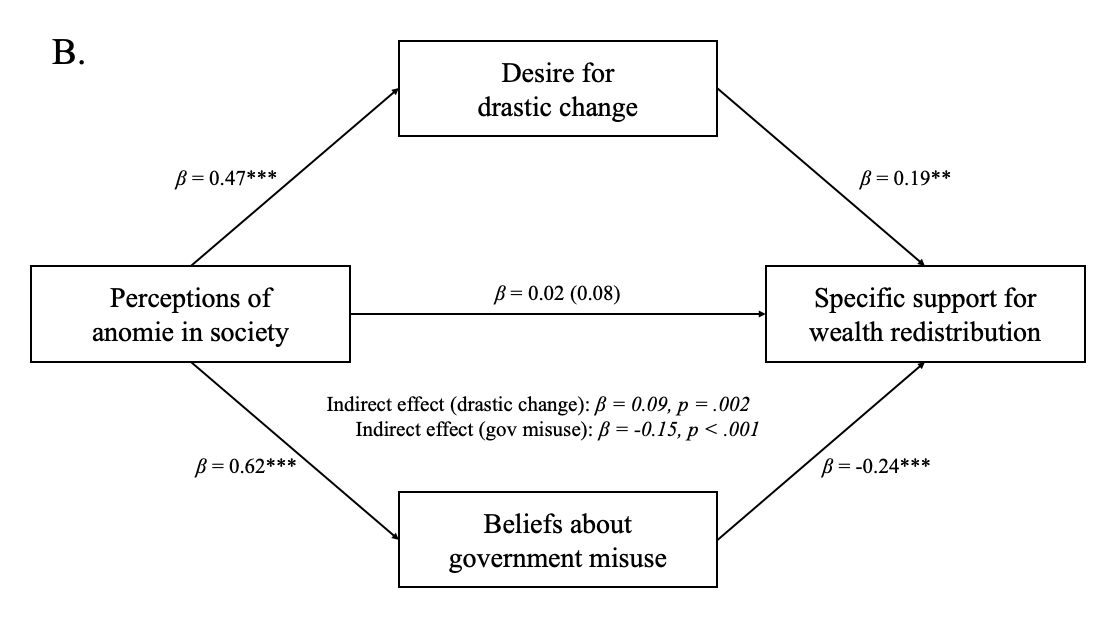


*Note:* Panel A depicts the relationship for general support for redistribution and Panel B depicts the relationship for specific support for redistribution. Standardized coefficients are given. Indirect effects were calculated for each of 5000 bootstrapped samples. The value outside parentheses on the c path is the total effect, and the direct effect is the value inside parentheses.

* *p* < .05 ** *p* < .01 *** *p* < .001

# Supplementary Materials 4 – Full Mediation Output for all Studies

**Study 1**

***Table S5.***

*Mediation Output for General Support for Redistribution*

| **Predictor → Outcome** | ***b*** | ***SE_boot_*** | ***z*** | ***p*** | ***β*** |
| --- | --- | --- | --- | --- | --- |
| **Paths to Mediators** |  |  |  |  |  |
| Anomie → Drastic Change (a₁) | 0.621 | 0.065 | 9.61 | < .001 | 0.416 |
| Anomie → Government Misuse (a₂) | 0.863 | 0.058 | 14.97 | < .001 | 0.546 |
| **Paths to General Redistribution** |  |  |  |  |  |
| Drastic Change → General Redistribution (b₁) | 0.617 | 0.053 | 11.65 | < .001 | 0.450 |
| Government Misuse → General Redistribution (b₂) | –0.476 | 0.056 | –8.47 | < .001 | –0.367 |
| Anomie → Redistribution (direct c′) | 0.064 | 0.087 | 0.74 | .462 | 0.031 |
| **Indirect Effects** |  |  |  |  |  |
| Indirect via Drastic Change (a₁b₁) | **0.383** | 0.049 | 7.87 | < .001 | 0.187 |
| Indirect via Gov. Misuse (a₂b₂) | **–0.411** | 0.055 | –7.43 | < .001 | –0.201 |
| **Total Effect (c)** | 0.037 | 0.089 | 0.42 | .677 | 0.018 |
| **R²** |  |  |  |  |  |
| General Redistribution | 0.272 |  |  |  |  |
| Drastic Change | 0.173 |  |  |  |  |
| Government Misuse | 0.298 |  |  |  |  |

***Table S6.***

*Mediation Output for General Support for Redistribution, Controlling for Political Orientation and Subjective Social Status*

| **Predictor → Outcome** | ***b*** | ***SE_boot_*** | ***z*** | ***p*** | ***β*** |
| --- | --- | --- | --- | --- | --- |
| **Paths to Drastic Change** |  |  |  |  |  |
| Anomie → Drastic Change (a₁) | 0.636 | 0.062 | 10.23 | < .001 | 0.426 |
| Political Orientation → Drastic Change | –0.210 | 0.032 | –6.59 | < .001 | –0.264 |
| Subjective Status → Drastic Change | 0.043 | 0.035 | 1.23 | .219 | 0.049 |
| **Paths to Government Misuse** |  |  |  |  |  |
| Anomie → Gov. Misuse (a₂) | 0.865 | 0.059 | 14.74 | < .001 | 0.547 |
| Political Orientation → Gov. Misuse | 0.234 | 0.027 | 8.57 | < .001 | 0.278 |
| Subjective Status → Gov. Misuse | –0.004 | 0.034 | –0.11 | .915 | –0.004 |
| **Paths to Support for General Redistribution** |  |  |  |  |  |
| Drastic Change → General Redistribution (b₁) | 0.424 | 0.051 | 8.38 | < .001 | 0.310 |
| Gov. Misuse → General Redistribution (b₂) | –0.243 | 0.051 | –4.79 | < .001 | –0.188 |
| Anomie → General Redistribution (direct c′) | –0.024 | 0.078 | 0.31 | .758 | –0.012 |
| Political Orientation → General Redistribution | –0.509 | 0.041 | –12.37 | < .001 | –0.467 |
| Subjective Status → General Redistribution | –0.001 | 0.041 | –0.02 | .982 | –0.001 |
| **Indirect Effects** |  |  |  |  |  |
| Indirect via Drastic Change (a₁b₁) | **0.270** | 0.039 | 6.84 | < .001 | 0.132 |
| Indirect via Gov. Misuse (a₂b₂) | **–0.210** | 0.045 | –4.68 | < .001 | –0.103 |
| **Total Effect (c)** | 0.036 | 0.069 | 0.51 | .607 | 0.017 |
| **R²** |  |  |  |  |  |
| Support for General Redistribution | 0.450 |  |  |  |  |
| Drastic Change | 0.243 |  |  |  |  |
| Government Misuse | 0.376 |  |  |  |  |

***Table S7.***

*Mediation Output for Specific Support for Redistribution*

| **Predictor → Outcome** | ***b*** | ***SE_boot_*** | ***z*** | ***p*** | ***β*** |
| --- | --- | --- | --- | --- | --- |
| **Paths to Mediators** |  |  |  |  |  |
| Anomie → Drastic Change (a₁) | 0.618 | 0.066 | 9.37 | < .001 | 0.415 |
| Anomie → Government Misuse (a₂) | 0.861 | 0.057 | 15.19 | < .001 | 0.545 |
| **Paths to Specific Redistribution** |  |  |  |  |  |
| Drastic Change → Specific Redistribution (b₁) | 0.487 | 0.061 | 8.02 | < .001 | 0.350 |
| Gov. Misuse → Specific Redistribution (b₂) | –0.476 | 0.062 | –7.71 | < .001 | –0.362 |
| Anomie → Specific Redistribution (direct c′) | 0.094 | 0.107 | 0.88 | .380 | 0.045 |
| **Indirect Effects** |  |  |  |  |  |
| Indirect via Drastic Change (a₁b₁) | **0.301** | 0.048 | 6.30 | < .001 | 0.145 |
| Indirect via Gov. Misuse (a₂b₂) | **–0.410** | 0.060 | –6.87 | < .001 | –0.198 |
| **Total Effect (c)** | –0.014 | 0.094 | –0.15 | .879 | –0.007 |
| **R²** |  |  |  |  |  |
| Specific Redistribution | 0.202 |  |  |  |  |
| Drastic Change | 0.172 |  |  |  |  |
| Government Misuse | 0.297 |  |  |  |  |

***Table S8.***

*Mediation Output for Specific Support for Redistribution, Controlling for Political Orientation and Subjective Social Status*

| **Predictor → Outcome** | ***b*** | ***SE_boot_*** | ***z*** | ***p*** | ***β*** |
| --- | --- | --- | --- | --- | --- |
| **Paths to Drastic Change** |  |  |  |  |  |
| Anomie → Drastic Change (a₁) | 0.634 | 0.063 | 10.14 | < .001 | 0.425 |
| Political Orientation → Drastic Change | –0.211 | 0.032 | –6.56 | < .001 | –0.266 |
| Subjective Status → Drastic Change | 0.047 | 0.035 | 1.33 | .183 | 0.054 |
| **Paths to Government Misuse** |  |  |  |  |  |
| Anomie → Gov. Misuse (a₂) | 0.864 | 0.058 | 14.83 | < .001 | 0.547 |
| Political Orientation → Gov. Misuse | 0.233 | 0.028 | 8.35 | < .001 | 0.278 |
| Subjective Status → Gov. Misuse | –0.002 | 0.034 | –0.05 | .958 | –0.002 |
| **Paths to Specific Redistribution** |  |  |  |  |  |
| Drastic Change → Specific Redistribution (b₁) | 0.280 | 0.055 | 5.08 | < .001 | 0.201 |
| Gov. Misuse → Specific Redistribution (b₂) | –0.225 | 0.053 | –4.27 | < .001 | –0.172 |
| Anomie → Specific Redistribution (direct c′) | –0.011 | 0.091 | 0.12 | .902 | –0.005 |
| Political Orientation → Specific Redistribution | –0.548 | 0.041 | –13.49 | < .001 | –0.497 |
| Subjective Status → Specific Redistribution | –0.024 | 0.042 | –0.58 | .562 | –0.020 |
| **Indirect Effects** |  |  |  |  |  |
| Indirect via Drastic Change (a₁b₁) | **0.177** | 0.039 | 4.58 | < .001 | 0.086 |
| Indirect via Gov. Misuse (a₂b₂) | **–0.195** | 0.047 | –4.13 | < .001 | –0.094 |
| **Total Effect (c)** | –0.028 | 0.074 | –0.39 | .700 | –0.014 |
| **R²** |  |  |  |  |  |
| Specific Redistribution | 0.405 |  |  |  |  |
| Drastic Change | 0.243 |  |  |  |  |
| Government Misuse | 0.375 |  |  |  |  |

**Study 2**

***Table S9.***

*Mediation Output for General Support for Redistribution*

| **Predictor → Outcome** | ***b*** | ***SE_boot_*** | ***z*** | ***p*** | ***β*** |
| --- | --- | --- | --- | --- | --- |
| **Paths to Mediators** |  |  |  |  |  |
| Anomie → Drastic Change (a₁) | 0.643 | 0.056 | 11.55 | < .001 | 0.481 |
| Anomie → Government Misuse (a₂) | 0.974 | 0.058 | 16.76 | < .001 | 0.623 |
| **Paths to General Redistribution** |  |  |  |  |  |
| Drastic Change → General Redistribution (b₁) | 0.288 | 0.067 | 4.31 | < .001 | 0.232 |
| Gov. Misuse → General Redistribution (b₂) | –0.310 | 0.062 | –4.96 | < .001 | –0.292 |
| Anomie → General Redistribution (direct c′) | 0.230 | 0.112 | 2.06 | .040 | 0.139 |
| **Indirect Effects** |  |  |  |  |  |
| Indirect via Drastic Change (a₁b₁) | 0.185 | 0.046 | 4.02 | < .001 | 0.112 |
| Indirect via Gov. Misuse (a₂b₂) | –0.302 | 0.065 | –4.67 | < .001 | –0.182 |
| Total Effect (c) | 0.114 | 0.084 | 1.35 | .178 | 0.069 |
| **R²** |  |  |  |  |  |
| General Redistribution | 0.093 |  |  |  |  |
| Drastic Change | 0.231 |  |  |  |  |
| Government Misuse | 0.388 |  |  |  |  |

***Table S10.***

*Mediation Output for General Support for Redistribution, Controlling for Political Orientation and Subjective Social Status*

| **Predictor → Outcome** | ***b*** | ***SE_boot_*** | ***z*** | ***p*** | ***β*** |
| --- | --- | --- | --- | --- | --- |
| **Paths to Drastic Change** |  |  |  |  |  |
| Anomie → Drastic Change (a₁) | 0.654 | 0.060 | 10.88 | < .001 | 0.490 |
| Political Orientation → Drastic Change | –0.039 | 0.035 | –1.10 | .272 | –0.048 |
| Subjective Status → Drastic Change | –0.006 | 0.038 | –0.15 | .882 | –0.007 |
| **Paths to Government Misuse** |  |  |  |  |  |
| Anomie → Gov. Misuse (a₂) | 0.959 | 0.056 | 17.19 | < .001 | 0.614 |
| Political Orientation → Gov. Misuse | 0.182 | 0.035 | 5.15 | < .001 | 0.191 |
| Subjective Status → Gov. Misuse | 0.097 | 0.036 | 2.68 | .007 | 0.103 |
| **Paths to General Redistribution** |  |  |  |  |  |
| Drastic Change → General Redistribution (b₁) | 0.251 | 0.061 | 4.14 | < .001 | 0.202 |
| Gov. Misuse → General Redistribution (b₂) | –0.138 | 0.059 | –2.34 | .019 | –0.130 |
| Anomie → General Redistribution (direct c′) | 0.147 | 0.102 | 1.43 | .152 | 0.089 |
| Political Orientation → General Redistribution | –0.409 | 0.044 | –9.27 | < .001 | –0.405 |
| Subjective Status → General Redistribution | –0.141 | 0.041 | –3.47 | .001 | –0.141 |
| **Indirect Effects** |  |  |  |  |  |
| Indirect via Drastic Change (a₁b₁) | 0.164 | 0.042 | 3.88 | < .001 | 0.099 |
| Indirect via Gov. Misuse (a₂b₂) | –0.132 | 0.058 | –2.30 | .021 | –0.080 |
| Total Effect (c) | 0.179 | 0.075 | 2.38 | .017 | 0.108 |
| **R²** |  |  |  |  |  |
| General Redistribution | 0.272 |  |  |  |  |
| Drastic Change | 0.236 |  |  |  |  |
| Government Misuse | 0.439 |  |  |  |  |

***Table S11.***

*Mediation Output for Specific Support for Redistribution*

| **Predictor → Outcome** | ***b*** | ***SE_boot_*** | ***z*** | ***p*** | ***β*** |
| --- | --- | --- | --- | --- | --- |
| **Paths to Mediators** |  |  |  |  |  |
| Anomie → Drastic Change (a₁) | 0.643 | 0.056 | 11.44 | < .001 | 0.481 |
| Anomie → Government Misuse (a₂) | 0.974 | 0.057 | 17.15 | < .001 | 0.623 |
| **Paths to Specific Redistribution** |  |  |  |  |  |
| Drastic Change → Specific Redistribution (b₁) | 0.251 | 0.078 | 3.22 | .001 | 0.189 |
| Gov. Misuse → Specific Redistribution (b₂) | –0.265 | 0.067 | –3.98 | < .001 | –0.233 |
| Anomie → Specific Redistribution (direct c′) | 0.132 | 0.124 | 1.06 | .288 | 0.074 |
| **Indirect Effects** |  |  |  |  |  |
| Indirect via Drastic Change (a₁b₁) | 0.161 | 0.053 | 3.06 | .002 | 0.091 |
| Indirect via Gov. Misuse (a₂b₂) | –0.258 | 0.066 | –3.89 | < .001 | –0.145 |
| Total Effect (c) | 0.036 | 0.091 | 0.39 | .696 | 0.020 |
| **R²** |  |  |  |  |  |
| Specific Redistribution | 0.058 |  |  |  |  |
| Drastic Change | 0.231 |  |  |  |  |
| Government Misuse | 0.388 |  |  |  |  |

***Table S12.***

*Mediation Output for Specific Support for Redistribution, Controlling for Political Orientation and Subjective Social Status*

| **Predictor → Outcome** | ***b*** | ***SE_boot_*** | ***z*** | ***p*** | ***β*** |
| --- | --- | --- | --- | --- | --- |
| **Paths to Drastic Change** |  |  |  |  |  |
| Anomie → Drastic Change (a₁) | 0.654 | 0.059 | 11.04 | < .001 | 0.490 |
| Political Orientation → Drastic Change | –0.039 | 0.035 | –1.09 | .274 | –0.048 |
| Subjective Status → Drastic Change | –0.006 | 0.038 | –0.15 | .883 | –0.007 |
| **Paths to Government Misuse** |  |  |  |  |  |
| Anomie → Gov. Misuse (a₂) | 0.959 | 0.056 | 17.18 | < .001 | 0.614 |
| Political Orientation → Gov. Misuse | 0.182 | 0.035 | 5.13 | < .001 | 0.191 |
| Subjective Status → Gov. Misuse | 0.097 | 0.036 | 2.67 | .007 | 0.103 |
| **Paths to Specific Redistribution** |  |  |  |  |  |
| Drastic Change → Specific Redistribution (b₁) | 0.212 | 0.071 | 2.98 | .003 | 0.159 |
| Gov. Misuse → Specific Redistribution (b₂) | –0.092 | 0.063 | –1.46 | .144 | –0.081 |
| Anomie → Specific Redistribution (direct c′) | 0.059 | 0.120 | 0.49 | .625 | 0.033 |
| Political Orientation → Specific Redistribution | –0.419 | 0.047 | –8.96 | < .001 | –0.388 |
| Subjective Status → Specific Redistribution | –0.126 | 0.049 | –2.57 | .010 | –0.117 |
| **Indirect Effects** |  |  |  |  |  |
| Indirect via Drastic Change (a₁b₁) | 0.138 | 0.048 | 2.88 | .004 | 0.078 |
| Indirect via Gov. Misuse (a₂b₂) | –0.088 | 0.060 | –1.46 | .144 | –0.050 |
| Total Effect (c) | 0.109 | 0.088 | 1.24 | .214 | 0.061 |
| **R²** |  |  |  |  |  |
| Specific Redistribution | 0.216 |  |  |  |  |
| Drastic Change | 0.236 |  |  |  |  |
| Government Misuse | 0.439 |  |  |  |  |

**Study 3**

***Table S13.***

*Mediation Output for General Support for Redistribution, Controlling for Perceptions of Inequality*

| **Predictor → Outcome** | ***b*** | ***SE_boot_*** | ***z*** | ***p*** | ***β*** |
| --- | --- | --- | --- | --- | --- |
| **Paths to Drastic Change** |  |  |  |  |  |
| Condition (High = 1, Low = 0) → Drastic Change (a₁) | 2.578 | 0.109 | 23.74 | < .001 | 0.768 |
| Inequality → Drastic Change | 0.028 | 0.024 | 1.18 | .240 | 0.037 |
| **Paths to Government Misuse** |  |  |  |  |  |
| Condition → Gov. Misuse (a₂) | 2.602 | 0.130 | 19.96 | < .001 | 0.706 |
| Inequality → Gov. Misuse | –0.019 | 0.028 | –0.65 | .513 | –0.022 |
| **Paths to General Redistribution** |  |  |  |  |  |
| Drastic Change → General Redistribution (b₁) | 0.692 | 0.071 | 9.82 | < .001 | 0.677 |
| Gov. Misuse → General Redistribution (b₂) | –0.275 | 0.060 | –4.57 | < .001 | –0.295 |
| Condition → General Redistribution (direct c′) | –0.478 | 0.209 | –2.28 | .022 | –0.139 |
| Inequality → General Redistribution | 0.029 | 0.033 | 0.86 | .389 | 0.037 |
| **Indirect Effects** |  |  |  |  |  |
| Indirect via Drastic Change (a₁b₁) | 1.784 | 0.204 | 8.74 | < .001 | 0.520 |
| Indirect via Gov. Misuse (a₂b₂) | –0.715 | 0.162 | –4.42 | < .001 | –0.208 |
| Total Effect (c) | 0.592 | 0.174 | 3.39 | .001 | 0.172 |
| **R²** |  |  |  |  |  |
| General Redistribution | 0.217 |  |  |  |  |
| Drastic Change | 0.606 |  |  |  |  |
| Government Misuse | 0.490 |  |  |  |  |

***Table S14.***

*Mediation Output for Specific Support for Redistribution, Controlling for Perceptions of Inequality*

| **Predictor → Outcome** | ***b*** | ***SE_boot_*** | ***z*** | ***p*** | ***β*** |
| --- | --- | --- | --- | --- | --- |
| **Paths to Drastic Change** |  |  |  |  |  |
| Condition → Drastic Change (a₁) | 2.578 | 0.107 | 24.11 | < .001 | 0.768 |
| Inequality → Drastic Change | 0.028 | 0.024 | 1.17 | .243 | 0.037 |
| **Paths to Government Misuse** |  |  |  |  |  |
| Condition → Gov. Misuse (a₂) | 2.602 | 0.132 | 19.70 | < .001 | 0.706 |
| Inequality → Gov. Misuse | –0.019 | 0.029 | –0.64 | .522 | –0.022 |
| **Paths to Specific Redistribution** |  |  |  |  |  |
| Drastic Change → Specific Redistribution (b₁) | 0.649 | 0.084 | 7.71 | < .001 | 0.592 |
| Gov. Misuse → Specific Redistribution (b₂) | –0.319 | 0.073 | –4.36 | < .001 | –0.319 |
| Condition → Specific Redistribution (direct c′) | –0.522 | 0.249 | –2.09 | .036 | –0.142 |
| Inequality → Specific Redistribution | –0.041 | 0.040 | –1.02 | .306 | –0.048 |
| **Indirect Effects** |  |  |  |  |  |
| Indirect via Drastic Change (a₁b₁) | 1.672 | 0.237 | 7.04 | < .001 | 0.455 |
| Indirect via Gov. Misuse (a₂b₂) | –0.829 | 0.198 | –4.20 | < .001 | –0.225 |
| Total Effect (c) | 0.320 | 0.190 | 1.68 | .092 | 0.087 |
| **R²** |  |  |  |  |  |
| Specific Redistribution | 0.151 |  |  |  |  |
| Drastic Change | 0.606 |  |  |  |  |
| Government Misuse | 0.490 |  |  |  |  |

# Supplementary Materials 5 – Results for all Studies by Anomie Subscale

**Study 1**

***Anomie in the Social Fabric***

***Table S15.***

*Correlation table (Study 1)*

|  | 1 | 2 | 3 | 4 | 5 |
| --- | --- | --- | --- | --- | --- |
| 1. Perceptions of anomie (social) | – |  |  |  |  |
| 2. Support for redistribution (general) | .03 | – |  |  |  |
| 3. Support for redistribution (specific) | .01 | .72*** | – |  |  |
| 4. Desire for drastic change | .28*** | .39*** | .30*** | – |  |
| 5. Beliefs about government misuse | .37*** | -.26*** | -.27*** | .20*** | – |

*Note*. * *p* < .05 ** *p* < .01 *** *p* < .001

***Figure S4.*** Parallel Mediation Analyses for Study 1


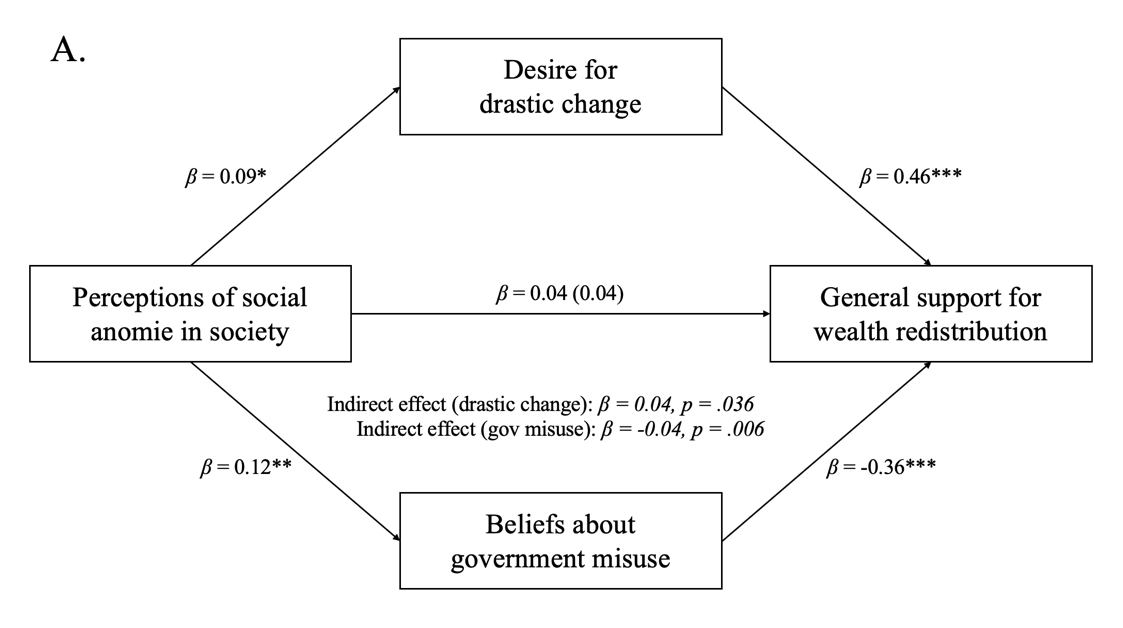


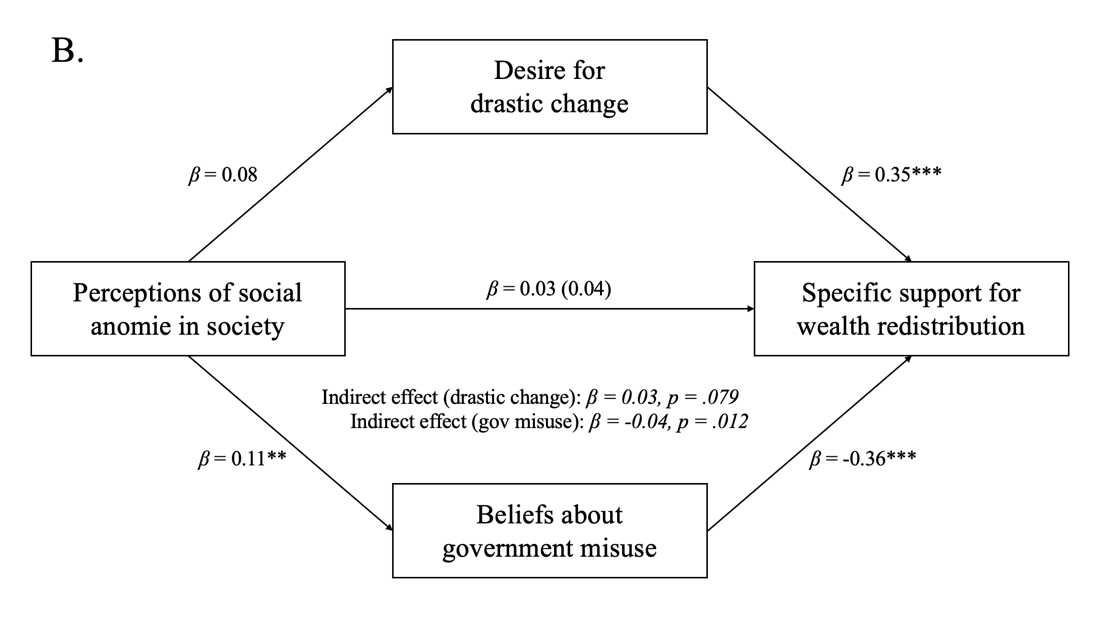


*Note:* Panel A depicts the relationship for general support for redistribution and Panel B depicts the relationship for specific support for redistribution. Standardized coefficients are given. Indirect effects were calculated for each of 5000 bootstrapped samples. The value outside parentheses on the c path is the total effect, and the direct effect is the value inside parentheses. Anomie in leadership is included as a covariate.

* *p* < .05 ** *p* < .01 *** *p* < .001

***Anomie in Leadership***

***Table S16.***

*Correlation table (Study 1)*

|  | 1 | 2 | 3 | 4 | 5 |
| --- | --- | --- | --- | --- | --- |
| 1. Perceptions of anomie (leadership) | – |  |  |  |  |
| 2. Support for redistribution (general) | <.01 | – |  |  |  |
| 3. Support for redistribution (specific) | -.02 | .72*** | – |  |  |
| 4. Desire for drastic change | .43*** | .39*** | .30*** | – |  |
| 5. Beliefs about government misuse | .56*** | -.26*** | -.27*** | .20*** | – |

*Note*. * *p* < .05 ** *p* < .01 *** *p* < .001

***Figure S5.*** Parallel Mediation Analyses for Study 1


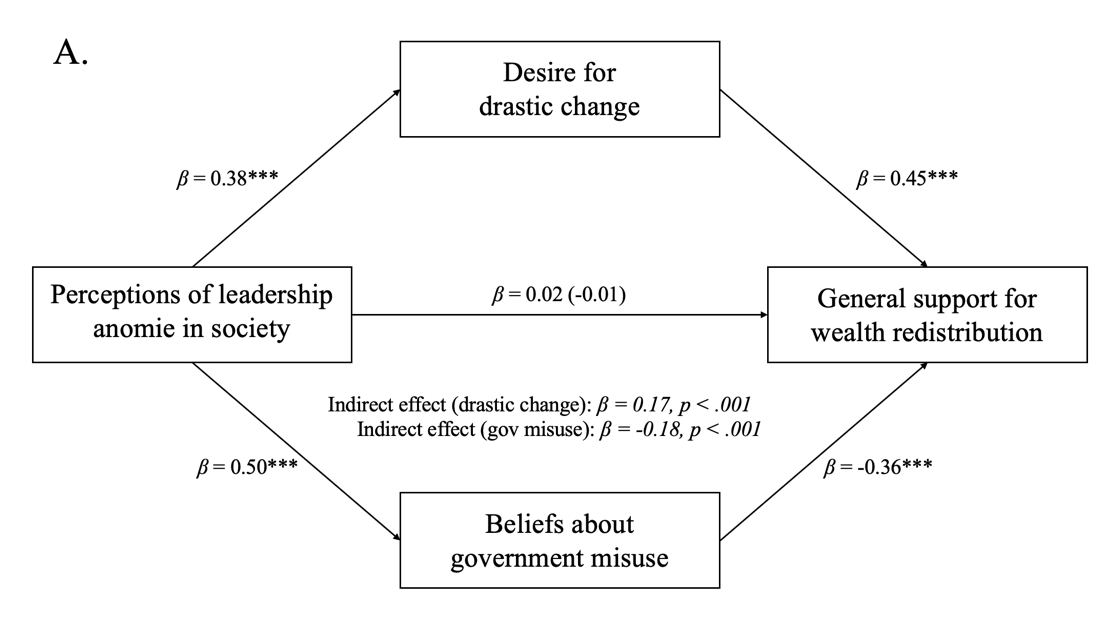


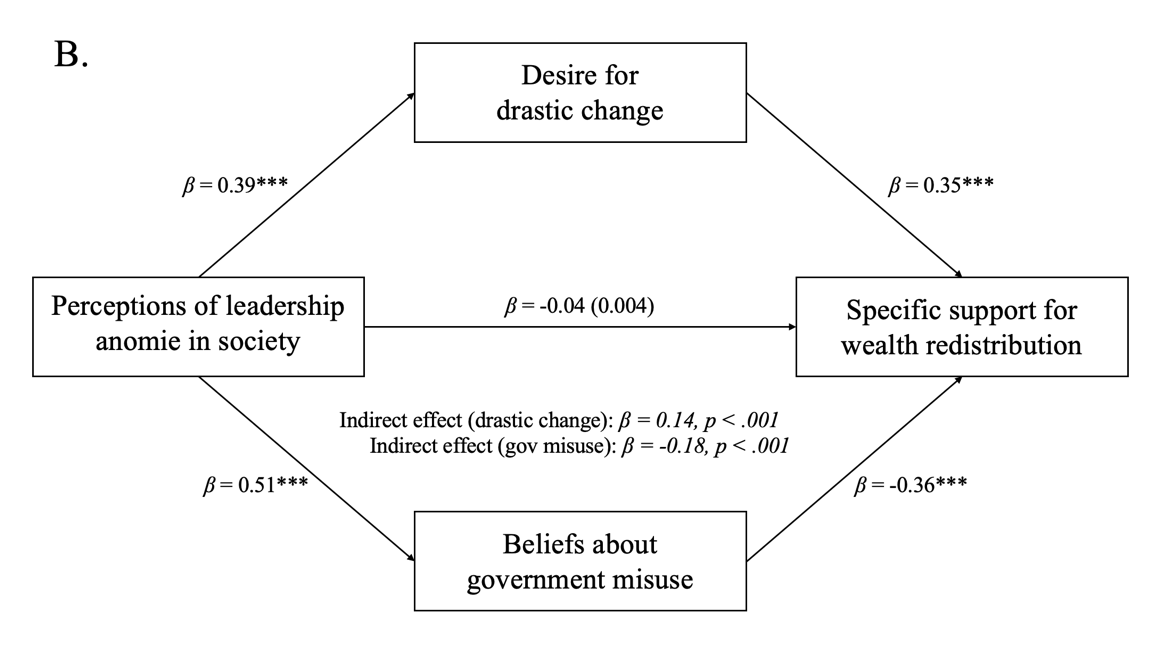


*Note:* Panel A depicts the relationship for general support for redistribution and Panel B depicts the relationship for specific support for redistribution. Standardized coefficients are given. Indirect effects were calculated for each of 5000 bootstrapped samples. The value outside parentheses on the c path is the total effect, and the direct effect is the value inside parentheses. Anomie in the social fabric is included as a covariate.

* *p* < .05 ** *p* < .01 *** *p* < .001

**Study 2**

***Anomie in the Social Fabric***

***Table S17.***

*Correlation table (Study 2)*

|  | 1 | 2 | 3 | 4 | 5 |
| --- | --- | --- | --- | --- | --- |
| 1. Perceptions of anomie (social) | – |  |  |  |  |
| 2. Support for redistribution (general) | .07 | – |  |  |  |
| 3. Support for redistribution (specific) | .01 | .64*** | – |  |  |
| 4. Desire for drastic change | .38*** | .20*** | .15** | – |  |
| 5. Beliefs about government misuse | .46*** | -.13** | -.12** | .34*** | – |

*Note*. * *p* < .05 ** *p* < .01 *** *p* < .001

***Figure S6.*** Parallel Mediation Analyses for Study 2


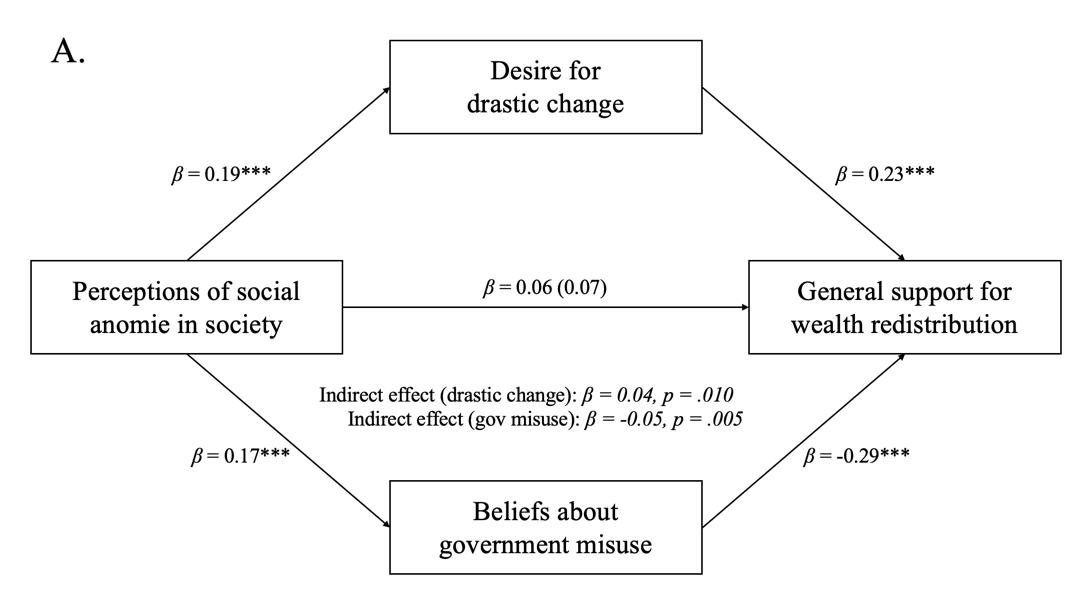


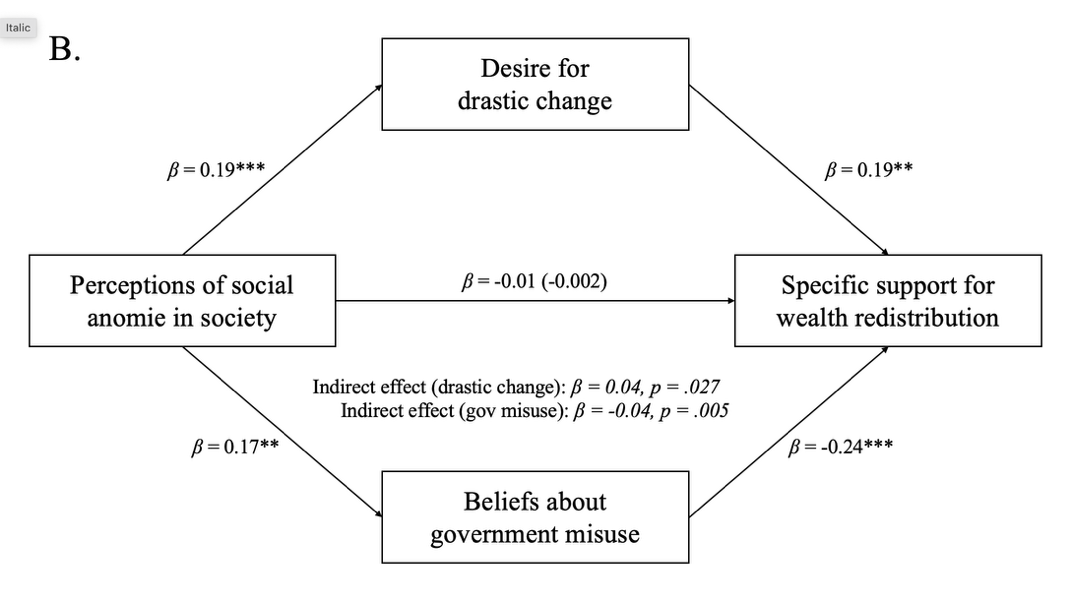


*Note:* Panel A depicts the relationship for general support for redistribution and Panel B depicts the relationship for specific support for redistribution. Standardized coefficients are given. Indirect effects were calculated for each of 5000 bootstrapped samples. The value outside parentheses on the c path is the total effect, and the direct effect is the value inside parentheses. Anomie in leadership is included as a covariate.

* *p* < .05 ** *p* < .01 *** *p* < .001

***Anomie in Leadership***

***Table S18.***

*Correlation table (Study 2)*

|  | 1 | 2 | 3 | 4 | 5 |
| --- | --- | --- | --- | --- | --- |
| 1. Perceptions of anomie (leadership) | – |  |  |  |  |
| 2. Support for redistribution (general) | .05 | – |  |  |  |
| 3. Support for redistribution (specific) | .03 | .64*** | – |  |  |
| 4. Desire for drastic change | .46*** | .20*** | .15** | – |  |
| 5. Beliefs about government misuse | .62*** | -.13** | -.12** | .34*** | – |

*Note*. * *p* < .05 ** *p* < .01 *** *p* < .001

***Figure S7.*** Parallel Mediation Analyses for Study 2


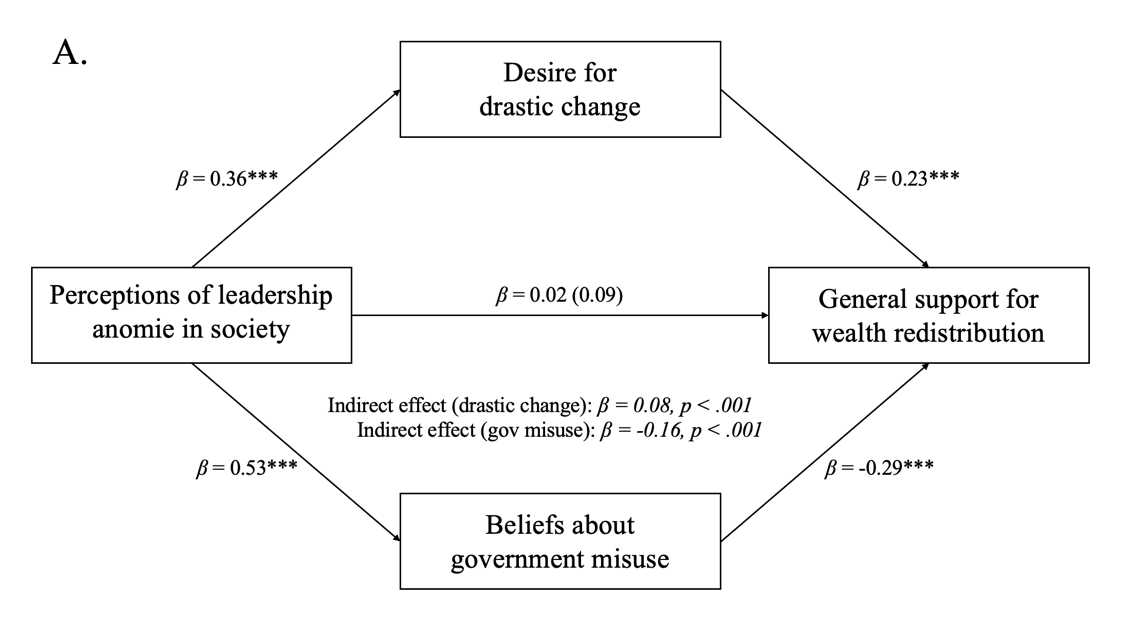


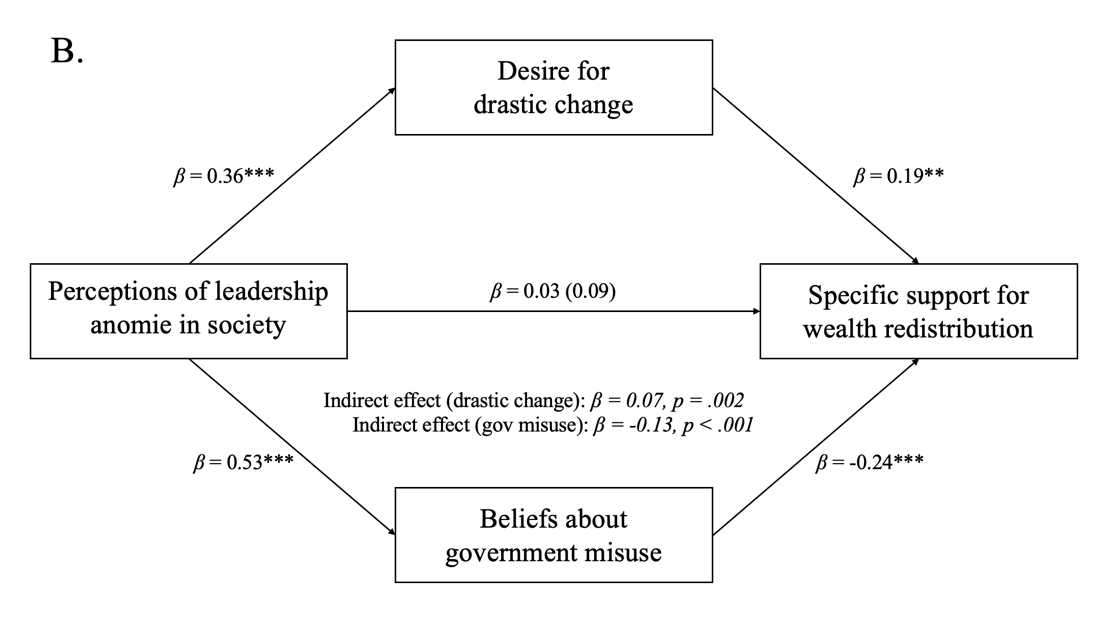


*Note:* Panel A depicts the relationship for general support for redistribution and Panel B depicts the relationship for specific support for redistribution. Standardized coefficients are given. Indirect effects were calculated for each of 5000 bootstrapped samples. The value outside parentheses on the c path is the total effect, and the direct effect is the value inside parentheses. Anomie in the social fabric is included as a covariate.

* *p* < .05 ** *p* < .01 *** *p* < .001

# Supplementary Materials 6 – Results for Unreported Study with Wording Error

This study was conducted prior to Study 2 in a UK sample (*N* = 617) with an identical methodology. However, a mistake was made for the item about government misuse of redistributed wealth: “Imagine the American government started taxing the rich with the intention of giving that money to the poor.” Here the UK participants were mistakenly asked about the US government rather than the UK government. Due to this error, we did not report the results in the main study. Key results can be seen below and the full preregistration, data and analysis can be seen on OSF (preregistration: <https://osf.io/8aygm/overview>; data: <https://osf.io/ayjzn/overview?view_only=02bf4c5b9d0f41cdbce296b06f1308b8>).

***Table S19.***

*Correlation table (Unreported Study)*

|  | 1 | 2 | 3 | 4 | 5 |
| --- | --- | --- | --- | --- | --- |
| 1. Perceptions of anomie | – |  |  |  |  |
| 2. Support for redistribution (general) | .06 | – |  |  |  |
| 3. Support for redistribution (specific) | .06 | .66*** | – |  |  |
| 4. Desire for drastic change | .42*** | .29*** | .24*** | – |  |
| 5. Beliefs about government misuse | .52*** | -.12** | -.10* | .27*** | – |

*Note*. * *p* < .05 ** *p* < .01 *** *p* < .001

***Figure S8.*** Parallel Mediation Analyses for Unreported Study


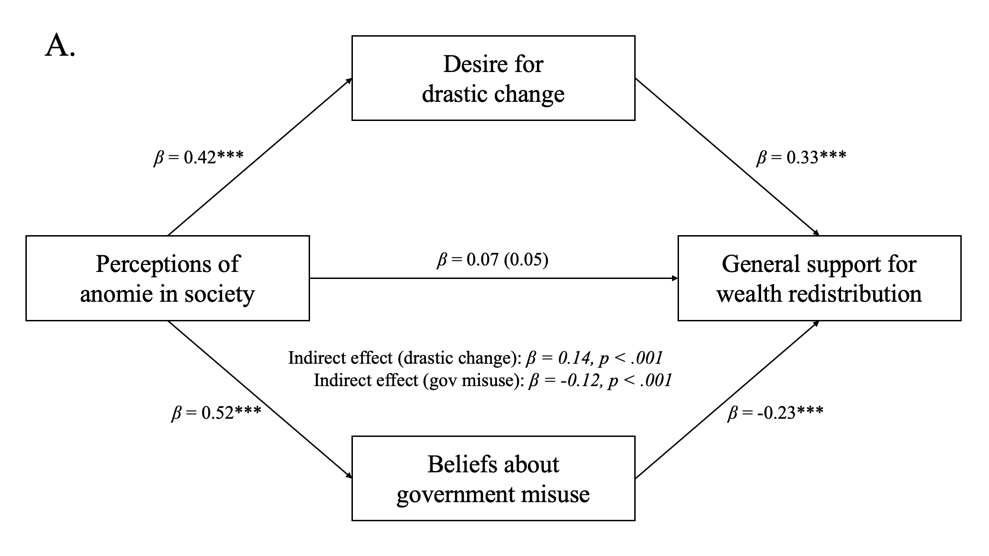

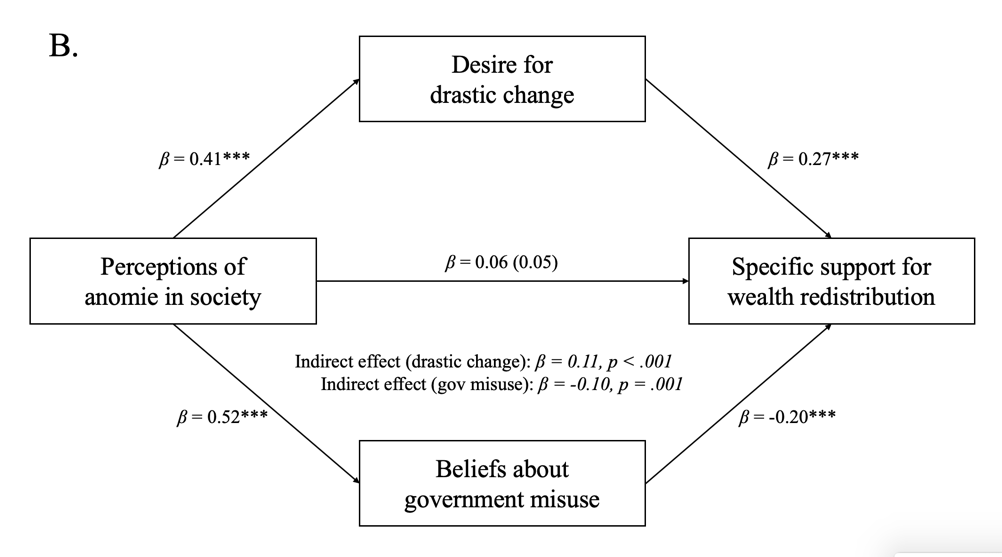


*Note:* Panel A depicts the relationship for general support for redistribution and Panel B depicts the relationship for specific support for redistribution. Standardized coefficients are given. Indirect effects were calculated for each of 5000 bootstrapped samples. The value outside parentheses on the c path is the total effect, and the direct effect is the value inside parentheses.

* *p* < .05 ** *p* < .01 *** *p* < .001

1. Manipulation checks were included the experimental manipulation not assessed in Study 1 (see Supplementary Materials 2 below for more detail). [↑](#footnote-ref-1)
2. Using real-world statistics was an ethical requirement, and we were not able to use deception. [↑](#footnote-ref-2)
3. Counts after removing participants who failed manipulation or attention checks as described in the main manuscript. [↑](#footnote-ref-3)
